# Ethnomedicinal Studies, Chemical Composition, and Antibacterial Activity of the *Mammea americana* L. Bark in the Municipality of Cértegui, Chocó, Colombia

**DOI:** 10.1155/2022/9950625

**Published:** 2022-01-19

**Authors:** Lina Mosquera-Chaverra, Manuel Salas-Moreno, José Marrugo-Negrete

**Affiliations:** ^1^Faculty of Engineering, Environmental Engineering Program, Universidad Tecnológica Del Chocó, Quibdó 270002, Colombia; ^2^Faculty of Engineering, Engineering Research Group, Fundación Universitaria Claretiana, Quibdó 270001, Colombia; ^3^Department Biology, Faculty of Naturals Science, Biosystematics Research Group, Universidad Tecnológica Del Chocó, Quibdó 270002, Colombia; ^4^Universidad de Córdoba, Carrera 6 No. 76-103, Montería, Córdoba 230003, Colombia

## Abstract

*Mammea americana* L. is a plant with diverse medicinal uses in the municipality of Cértegui, Chocó, Colombia. This research characterized the ethnomedicinal, chemical, and antibacterial activities of the bark of *Mammea americana*. Through interviews and semistructured surveys with the community, its ethnomedicinal uses were determined. Compounds present in the bark extract were identified and quantified by gas chromatography-coupled to mass spectrometry (GC-MS), and a qualitative analysis was performed by preliminary phytochemistry. Antibacterial activity and minimum inhibitory concentration (MIC) were carried out by agar diffusion and dilution methods, respectively, using ethanolic and aqueous extracts. Ethnomedical data showed that the bark is used to treat 14 conditions, the most representative being gallstones, prostate inflammation, and malaria. Preliminary phytochemical analyses showed the existence of several secondary metabolites such as tannins, alkaloids, flavonoids, triterpenes and/or steroids, quinones, and saponins. A total of 29 compounds were identified; the most abundant were ethyl 5-oxo-4-(p-toluidine)-2,5-dihydro-3-furancarboxylate, phenol, 4,4′,4″-ethylidynetris, nerolidol, 19-hydroxy-13-epimanoyl oxide, *α*-elemene, and *δ*-cadinene. The results showed remarkable antibacterial activity of the ethanolic extract (20 mg/ml) against *Staphylococcus aureus* (22.6 mm) and *Escherichia coli* (19.6 mm) and of the crude water extract (20 mg/ml) against *Staphylococcus aureus* (18.5 mm) and *Escherichia coli* (12.4 mm). The strongest MIC was for the ethanolic extract with values of 0.357 and 0.897 mg/ml against *S. aureus* and *E. coli* strains, respectively, while in the aqueous extract, *S. aureus* (3.99 mg/ml) and *E. coli* (4.3 mg/ml) were recorded. It is assumed that the compounds identified in this study could be responsible for the antibacterial activity of the species, as well as the relationship of the identified compounds and metabolites with the ethnomedical uses given by the community, providing a scientific and traditional basis for its different traditional medical uses.

## 1. Introduction

There is a pressing need to develop new and innovative antimicrobial agents. Volatile secondary metabolites are one of the main antimicrobial compounds in plants [1] because the number of resistant microorganisms has increased and is becoming one of the greatest threats to global health affecting people of any age and anywhere in the world [2]. The WHO data show that 25% of deaths worldwide are caused by bacterial infections due to the difficulty to identify the microorganism for treatment [3].

Plants throughout the development of science have played a fundamental role in the search for compounds or extracts with medicinal characteristics. Herbal therapeutic solutions play an essential role in the primary healthcare of 80% of the world's population [4]. The plants produce secondary metabolites, which are an important source of drugs, especially in traditional medicine [5]. Secondary metabolites are responsible for multiple biological activities and may contribute to the treatment of diseases of bacterial origin such as gastroenteritis, diarrhea, urinary tract infections, sepsis, pneumonia, neonatal meningitis, and peritonitis [6–8].

*Mammea americana* L. (family Calophyllaceae) is a tree native to the West Indies and northern South America. It is cultivated in the Bahamas and, on a smaller scale, in Venezuela, Guyana, Suriname, French Guiana, Ecuador, and northern Brazil. In Colombia, it is found in humid forests, such as the Department of Chocó, and in temperate and warm zones, such as the Department of Cundinamarca. The *Mammea americana* L. tree can reach up to 25 m in height. Its trunk and branches are covered with a dark brown, rough bark. The fruit is a big flattened spherical dupa that can reach 10–25 cm in diameter wrapped in a thick brown skin with a leathery appearance [9]. It is commonly known as mamey, mamey apple, mamey de Santo, South American apricot, abricó-do-pará, abricó, Santo Domingo apricot, mamey amarillo, mamey de Cartagena, mata serrano, zapote mamey, and zapote de Santo Domingo [10, 11].

This plant was included in Tramil, a comprehensive study of the medicinal plant resources of the Caribbean [12]. All its parts can be used for the treatment of various diseases, with differences in medicinal preparations depending on the country [13]. To these plants are attributed properties such as antioxidants, antidiabetic, antimalarial, fungal infections, eczema, skin diseases, treatment of periodontal diseases, indications as convulsive tonic, parasitic, against stomach pain, anthelmintic, and digestive diseases, and antibacterial [14–20]. Studies have shown the potential of *Mammea americana* bark to treat gastrointestinal disorders, including gastric ulcers in ethanolic, methanolic, and dichloromethane extracts [18]. Antimicrobial activity performed on crude ethanolic extract of endophytic fungi of *M. americana* showed that it has antimicrobial effects on *S. aureus* and *E. coli* [21]. Similarly, it has been reported that the ethanolic extract of the leaves and bark of this plant also possesses antibacterial activity against *S. aureus* [22], while its seeds were shown to have inhibitory activity for *E. coli*, *Pseudomonas* spp., and *Salmonella* ssp. [23].

Information on the phytochemicals present in *M. americana*; it was found that the ethanol extract of the plant contained alkaloids, coumarins, phenols, tannins, quinones, and flavonoids in leaves and bark [22] and terpenoids, phenolic compounds, and flavonoids in the pulp of their fruit [20, 24, 25].

The objective of this investigation is the first to determine the medicinal uses that the bark of these plants presents through a set of cultural and central knowledge of the community, for the different inhabitants of the municipality of Cértegui. Subsequently, we relate the medicinal uses with an analysis of the secondary metabolites identified by preliminary phytochemical analysis and gas chromatography-coupled mass spectrometer (GC/MS). Finally, the antibacterial activity of the extract from *M. americana* bark will be evaluated against four bacterial strains, two Gram-positive and two Gram-negative. In this way, we intended to establish a relationship between the medicinal uses granted by the community, the antibacterial activity of the extract, and the identified secondary metabolites, which is important to scientifically justify traditional knowledge, giving added value to these plants.

## 2. Materials and Methods

### 2.1. Study Area

**Figure 1:**
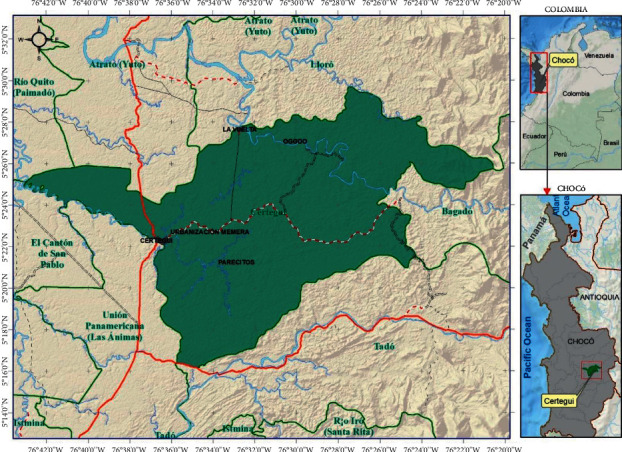
Location municipality of Cértegui. Source: Cértegui City Hall, 2020.

This research was performed in the municipality of Cértegui, Department of Chocó, Colombia (Figure 1). It has an area of 342 km^2^, located at 5°41′41″ north latitude and 76°39′40″ west longitude, with an altitude of 43 m above the sea level. The annual precipitation is 7000 mm; its average temperature is 28°C. It presents a topography characterized by low hills, according to the classification system reported by Rangel [26]. It is bordered to the north by the municipalities of Atrato and Lloró, to the south by the municipalities of Unión Panamericana and Tadó, to the east by the municipalities of Lloró and Bagadó, and to the west by the municipalities of Río Quito and Cantón del San Pablo [27].

### 2.2. Ethnomedical Phase and Data Analysis

The ethnomedicinal information of the cortex of the species *Mammea americana* L. was obtained through interviews and semistructured surveys. The most relevant data taken into account by the interviewees were sex, age, and occupation to ensure that the medicinal use of the plant species came from the traditional knowledge of the community. The medicinal information of the plant was validated as in the studies reported in the literature. The data obtained were processed in Microsoft Excel, 2016.

The plant material (bark) was pressed and transferred to the herbarium of the Universidad Tecnológica del Chocó Diego Luis Córdoba for taxonomic identification, presenting registration number 15142.

### 2.3. Preparation of the Extract

The bark was washed and disinfected with a 2% NaClO solution. It was air-dried at room temperature for seven days. Then, it was submitted to a temperature of 40°C, in an oven with circulating air (Memmert 854, Schwabach, Germany), always verifying humidity loss (three days approximately). The cortex was ground to a fine powder and then weighed.

The crushed and weighed plant material was then subjected to cold maceration for three days in 96% ethanol. Successive concentrations of this extract were made at reduced pressure in a digital rotary evaporator (model R-124, vacuum controller V-800, Buchi) at 40°C to obtain an ethanolic extract, which was stored at 4°C until the phytochemical screening was performed.

### 2.4. Phytochemical Screening

Qualitative tests were determined at the Natural Marine Products Laboratory of the University of Antioquia (Antioquia, Colombia). The qualitative identification of tannins, flavonoids, quinones, coumarins, saponins and sapogenins, and triterpenes and/or steroids was performed following the methodology of Bilbao [28, 29].

### 2.5. Analysis by GC/MS

100 g of plant material was sent to the Laboratory of Instrumental Analysis of the Universidad Nacional de Colombia (Medellin Headquarters, Medellin, Colombia). The extraction of the volatile compounds of the vapor phase of the sample was performed through the technique of solid-phase microextraction (SPME), with monitoring in the vapor phase (head space), using a fused silica fiber coated with polydimethylsiloxane–divinyl benzene of 65 *μ*m thickness (PDMSDVB-65 *μ*m, Sigma) [30].

The chromatographic analysis was performed in a 6890 Series Plus Gas Chromatograph (Agilent Technologies, USA), coupled to a mass selective detector (Agilent Technologies MSD 5973, USA). The column used for the analysis was DB-5MS (5% phenyl poly (methylsilane), 30 m × 0.32 mm *x* 0.5 *μ*m, Agilent); injection was performed with the SPME device. Identification of secondary metabolites was established based on their mass spectra (EI, 70 eV). The databases used were NIST98.l, NIST02.L, and NIST5a.L from the laboratory in question. Three biological replicates were used, and all analyses were done in duplicate.

### 2.6. Antibacterial Activity Assessment

Antibacterial activity was evaluated using the agar diffusion method [31]. In this study, four bacterial strains were tested, two Gram-positive bacteria (*Bacillus subtilis* ATCC 6633 and *Staphylococcus aureus* ATCC 25923) and two Gram-negative bacteria (*Escherichia coli* ATCC 25922 and *Pseudomonas aeruginosa* ATCC 13076).

The bacterial strains were first cultured in the Mueller-Hinton medium at 37°C for 24 hours before planting on nutrient agar. The inoculums of the bacteria used were stained on Mueller-Hinton agar plates using a sterile swab. The dilutions of the aqueous and ethanolic extracts used were 20, 10, 5, 2.5, and 0.5 mg/ml.

The paper discs used were 5 mm, with the amount of extract in each disc 20 *μ*l, the thickness of the agar 4 mm, and were placed on the surface of the agar, pressing gently on the surface of the agar with sterile forceps. Before incubating at 37°C, the plates were refrigerated at 4°C for three hours to allow the oil to diffuse. The positive control was streptomycin sulfate 10 *μ*g/ml, and the negative control was TSA (trypticase soy agar). After the incubation period (24 hours), the diameters of the inhibition halos produced were measured. All tests were performed in triplicate, and the means were calculated as final results. To determine the sensitivity of the *Mammea americana* L. bark extract, it was classified by the diameter of the inhibition halos, following the methodology: not sensitive (−) for diameters below 8 mm, sensitive (+) for diameters between 9 and 14 mm, very sensitive (++) for diameters ranging from 15 to 19 mm, and extremely sensitive (+++) for diameters above 20 mm [32].

### 2.7. Determination of Minimum Inhibitory Concentration (MIC) of Crude Extracts

The MIC was performed on the ethanolic and aqueous extracts that showed bactericidal activity in the antibacterial activity evaluation test, i.e., the minimum concentrations of the extract at which no growth was observed in the solid medium. The minimum inhibitory concentration of the crude extracts of the bark of *M. americana* L. was determined by the agar dilution method. The culture media after preparation were sterilized by autoclaving. The sterilized culture media were allowed to cool to 50°C, and 20 ml of molten agar was added to test tubes containing 2.0 ml of different concentrations (aqueous extract, ethanol extract, and control). The concentrations used ranged 0.0020–5.0 mg/ml. Suspensions of the respective bacteria having density adjusted to McFarland 0.5 turbidity units (1.5 × 10^8^ CFU/ml) were inoculated onto a series of agar plates using a standard loop. The dilution series was prepared using broth microdilution (1 : 100). Incubation was performed at 37°C for one day. The experiments were performed in duplicate and repeated three times (*n* = 3). The lowest concentration of the crude extract of *M. americana* was expressed as MIC in mg/ml [32].

### 2.8. Data Analyses

The experimental data were expressed as the mean of three repetitions and their standard deviation. Data analysis was performed using GraphPad Prism 9 statistical program. Analysis of variance (ANOVA) was used with Tukey's multiple comparisons test, with a 95% confidence level. A *p* value of less than 0.05 was considered statistically significant.

## 3. Results

### 3.1. Ethnomedical Phase

Traditional applications of the plant species by community residents in medicine show that the bark is used to treat diseases related to gallstones and kidney stones, prostate, and malaria, among others; it is also used as a laxative.  Table 1 presents the ethnomedicinal information and demographic characteristics of the people surveyed, which were 110, including men and women, with women being the ones who know the most about the plant. The age of the participants varied between 21 and 80 years (mean age 50.5 ± 17.4 years), with the highest range being between 41–50 years and 51–60 years. Regarding the occupation or economic activity they performed, most of them stated that they are dedicated to mining and in the second instance to the exploitation of wood.

### 3.2. Phytochemical Screening

The presence of secondary metabolites was performed according to the individual qualitative rather test for each group.  Table 2 provides the results of the phytochemical screening performed on the ethanolic extract of the bark of *M. americana* L., where a high diversity of secondary metabolites is observed in this species, such as tannins, alkaloids, flavonoids, triterpenes and/or steroids, quinones, saponins, and sapogenins. It can be said that these bioactive components could be responsible for the bioactivity demonstrated in the ethanol extract.

### 3.3. Volatile Compounds in *M. americana*

**Figure 2:**
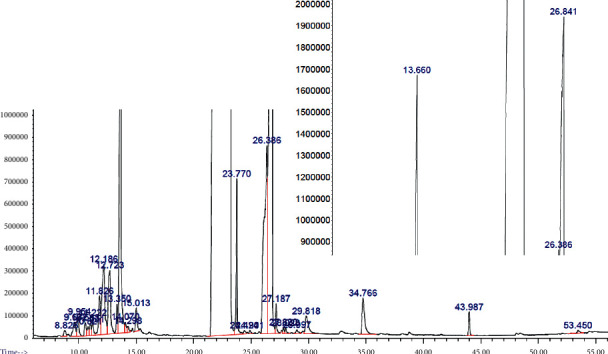
Chromatogram of the compounds present in the ethanol extract of Mammea Americana bark.

GC-MS analysis was performed on the extract to identify specific bioactive compounds that could be associated in part with the different medicinal uses given by the community and with the antibacterial activity evaluated in this study. A total of 29 volatile compounds were identified in a concentration of more than 0.01%, representing 100% of the total volatile compounds (Table 3), while the chromatogram is shown in Figure 2. Thus, the classes of secondary metabolites identified were sesquiterpenes (4.3%), oxygenated sesquiterpenes (5.18%), alkaloids (75.96%), unsaturated fatty acid (0.21%), monoterpene (0.05), steroids (4.28%), triterpenes (0.21), phenylpropanoid (0.26%), and phenolic compounds (9.83%). The individual compounds with the highest relative amount in the sample are ethyl 5-oxo-4-(p-toluidino)-2,5-dihydro-3-furancarboxylate (73.7%), 1,1,1-tris (4-hydroxyphenyl) ethane (9.83%), nerolidol (4.49%), 19-hydroxy-13-epimanoyl oxide (4.28%), *α*-elemene (1.38%), and *δ*-cadinene (1.13%).

A compound by itself or in combination with others likely contributes to the bioactivity present in the extract.

### 3.4. Antibacterial Activity Assessment

The antibacterial activity of *M. americana* L. against bacterial strains was qualitatively evaluated through the diameter of the inhibition zone. The antibacterial activity was analyzed in ethanolic extract, and then, it was proceeded to evaluate its general bioactivity in aqueous extract (crude water), in order to simulate the traditional preparation used by the community of the municipality of Cértegui.

The antibacterial activity of *M. americana* L. against the bacterial strains tested was qualitatively evaluated through the diameter of the inhibition zone. The results indicate that the ethanolic extract from the bark of the plant exhibited antibacterial activity against *Staphylococcus aureus* and *Escherichia coli* strains but in a concentration-dependent manner. The positive control, streptomycin control, was optimal in this test. The highest inhibition zone diameters were recorded for the *S. aureus* strain (22.6 mm), showing the strong activity of the plant bark which has extremely sensitive activity against this strain. The concentrations evaluated in this strain presented diameter ranges between sensitive and extremely sensitive; on the other hand, the *E. coli* strain inhibition diameter ranged from nonsensitive to very sensitive as the extract concentration decreased, registering a value of 6.4 mm for the minimum concentration used. The results of this activity are presented in Table 4.

### 3.5. Minimum Inhibitory Concentration (MIC) of the Crude Extracts

The effectiveness of ethanolic and aqueous extracts of *M. americana* was evaluated using the minimum inhibitory concentration (MIC) in those extracts that showed significant antibacterial activities, i.e., those extracts that showed halos greater than 8.0 mm in the zone of inhibition. Bacteria were added to diluted ethanolic and aqueous extracts at concentrations ranging from 0.0503 mg/ml to 0.191 mg/ml (Table 5). The strongest MIC was for the ethanolic extract with values of 0.0503 and 0.074 mg/ml against *S. aureus* and *E. coli* strains, respectively. The results also showed that the most significant MIC was against *S. aureus*.

## 4. Discussion

### 4.1. Ethnomedical Information

In general, plant species are used in ethnomedical practices for the treatment of different conditions; however, a large part of the world's plant population remains unexplored [33]. *M. americana* is used within traditional medicine in the municipality of Certeguí, Department of Chocó, Colombia, for the treatment of different diseases. Within the population surveyed in this study, there was a predominance of women over men to collect information on the plant; this is because the work activities of most women are in the morning hours (artisanal mining), unlike men who work all day, not coinciding with the interview schedule that was in the afternoon hours. Interview and survey data showed that the most representative medicinal uses of the plant were for gallstone pain, prostate inflammation and pain, and malaria. Most of the people surveyed made an aqueous extract from the bark of *M. americana* for several days; the patient took this extract three times a day. On the other hand, to treat a symptom such as toothache, the bark of the plant is used in the form of poultice. The community makes the poultice by pounding the bark and making a semisolid aqueous extract, which is applied to the cheek of the affected person. Similar research conducted on *M. africana* and *M. siamensis* reports that these are used in folk medicine to treat diabetes, arterial hypertension, hyperglycemia, and infectious diseases such as stomach pain, skin diseases, rheumatic pains, stomach, and cervical cancer, used in infusion or poultice [34–36].

The traditional knowledge of medicinal plants held by the respondents has been passed down from generation to generation by traditional doctors. We do not have historical data related to the placement of the *M. americana* L. to treat the different affections mentioned in Table 1; however, the results allowed us to affirm that the people surveyed acquired knowledge about this plant by the teaching of their parents or grandparents. According to Manya et al. [37], ethnomedicinal practice can be performed by men or women and is mainly practiced by adults and older people. On the other hand, [38] express that the family context is common in herbalists or ethnomedical practitioners, recognizing this knowledge as a family legacy, where the family is considered a social unit that subsists in an everyday life in which customs, norms, and practical knowledge such as traditional medicine are transmitted. However, some also state that their knowledge comes from years of experience and from their natural space where they have related with coworkers, capturing the knowledge of others who use medicinal plants [39].

Similar studies have been conducted in Chocó where plants such as *Jacaranda caucana* Pittier (Gualanday) (family Bignoniaceae) are used as infusion and/or plaster by the communities of Tutunendo and Villa Conto for rheumatoid arthritis pain, *Columnea cruenta* Morley (chicken's blood) (family Gesneriaceae) is used in the form of infusion by the community of Novita (Region of San Juan) for the relief in renal affections, and *Besleria barclayi* L.E. Skog (Sparkling) (family Gesneriaceae) is used by the community of Pacurita (Quibdó) as a disinfectant agent, for menstrual ailments and problems of sexual impotence [40, 41].

Since ancient times, humans have been using traditional medicine, either through herbal preparations, to treat, manage, and cure various ailments. Therefore, this type of medicine provides a solid foundation for the discovery of new agents against health-threatening pathogens [42].

### 4.2. Phytochemical Screening

The medicinal properties of this plant are associated with the biological activities of one or a group of its compounds. Characterization of the active compounds present in the ethanolic extract of the bark was performed by qualitative phytochemical screening. Some of the secondary metabolic chemicals have matched the report of Rodriguez et al. [22], alkaloids, tannins, quinones, and flavonoids. In this investigation work, the presence of alkaloids, phenols, and tannins, quinones, and flavonoids in the extract of its leaves was also reported. Similarly, the studies of [18, 20, 23] indicated that flavonoids, alkaloids, saponins, and tannins were present in the fruit of this plant. The presence of alkaloids, flavonoids, saponins, and quinones in the seed has also been determined [23]. In general, phytochemical investigations of various parts of *M. americana* have revealed the presence of flavonoids, terpene compounds, and volatile compounds [43]. Phytochemical studies in *Mammea* species have reported the presence of triterpenes and pentacyclic steroids [44]. Flavonoids, saponins, and alkaloids are reported to be present in the aqueous and ethanol extracts of *M. suriga* root bark [45].

Tannins, alkaloids, flavonoids, and triterpenes have been reported to have antibacterial and anti-inflammatory activities [46–49], analgesic properties occur in alkaloids and triterpenes [50, 51], and anticancer properties in saponins, flavonoids, and quinones [49, 52, 53].

Community surveys and interviews conducted in this research could attribute analgesic, anti-inflammatory, antimalarial, antibacterial, antianemic, anthelmintic, or laxative properties. However, it is necessary to determine other biological activities in future studies to scientifically validate the community's knowledge. In agreement with the above, other studies have shown that this plant has antibacterial, antiulcerogenic, and antimalarial activities in the leaves and bark [14, 18, 22, 52], antioxidant activity in the fruit pulp [24], and antitumor activity in the oil of its seeds [53].

Although phytochemical analysis provides important qualitative data, it is not a reliable tool to establish a relationship between ethnomedical use and secondary metabolites present in the plant. From this point of view, GC/MS was used, which provides more precise information on the constituents of the plant bark.

### 4.3. Quantification of Identified Compounds by GC/MS

The compounds identified within their chemical structure present carbon chains or rings, which make them not very soluble in water, giving them lipophilic characteristics, and they could be identified by GC-MS.

In our study, the alkaloids were the most abundant compounds. Alkaloids are medically known as stimulants, local anesthetics, antibacterials, antihypertensives, and analgesics [54], relating it to the different ethnomedicinal uses of our research (menstrual cramps, toothache, abdominal pain, and pain produced by gallstones and kidney stones). Interestingly, among the compounds identified, the alkaloid ethyl 5-oxo-4-(p-toluidino)-2,5-dihydro-3-furancarboxylate was the main compound in the bark of the plant, which is strange, since there is no information about this compound. However, to establish a relationship between the biological activities of the compound and the ethnomedicinal information, the alkaloids of the p-toluidine ring, such as p-toluidine 4-(3-aminopropyl) morpholino and p-toluidine methylamino, possible antimalarials, were taken as reference [55].

Malaria was one of the diseases present in the survey of this research, which could be related to this type of compounds. Similarly, the alkaloids 5,6,7-trimethoxy-2,3-dihydro (2,3-b) quinoline, pyrido[2,3-b]pyrimido [4,5-d]thiophen-4 (3H)-one, 3-amino-9-methoxymethyl-2,7-dimethyl, and 9-phenylcarbazole also have no reported biological activity; however, the main rings of these compounds have antimalarial, anti-inflammatory activities [54, 56, 57], antimicrobial, and anticancer activities [58, 59] and possible antitumor agents [60], respectively. Another alkaloid identified was atherospermidine, which reports relaxing activity in uterine contractions, as well as antioxidant activity in plants such as *Artabotrys maingayi* and *A. elliptica*, respectively [61, 62], allowing to relate this activity with the medicinal use given by the community concerning conditions such as menstrual cramps.

Phenolic compounds have been implicated as important antioxidants in natural products, since they possess an ideal structural pattern for free radical stabilization [63]. Among phenolic compounds, phenol 4,4′,4″-ethylidynetris was identified, which reports antiestrogenic activity in vivo [64]. Estrogens are considered one of the main risk factors for the development of breast and uterine cancer [65].

Terpenoids were the group with the highest number of compounds identified (17) (Table 3). Terpenes are known for their antimicrobial or bactericidal, analgesic, and anti-inflammatory activities [48, 50, 66]. Nerolidol is a compound with antimalarial, antiparasitic, and antileishmanial activities [67–69], which could be related to the diseases reported by the community in this study, such as malaria and intestinal parasites. Other compounds identified were *α*-elemene and *δ*-cadinene, which have anticancer activity in both in vitro and in vivo models [70] and antimicrobial, antifungal, and larvicidal activities against malaria, dengue, and filariasis mosquitoes [71–73], respectively. Squalene has activity against high cholesterol, is an antioxidant, and inhibits the development of several tumors [74, 75]. Although most of the compounds identified within the terpenes group present relative amounts lower than 1%, they could contribute to different activities considering they can act in synergy with other compounds.

The labdane-type diterpene compound, 19-hydroxy-13-epimanoyl oxide, was identified with a relative amount of 4.28%. This compound has been revealed to have antimicrobial and antifungal activities in plants such as *Cistus creticus* L. (Cistaceae) [76]; the essential oil from the leaves of this plant has, among its main constituents, 13-epimanoyl oxide, showing strong microbial activity against strains of *S. aureus* and *Bacillus subtilis* [77], matching those reported for the antibacterial activity of *M. americana*, compared to the strong activity exhibited by *S. aureus*. Moreover, this activity coincides with the data provided by the studied community for the treatment of intestinal parasites.

Finally, groups such as fatty acids and phenylpropanoids only present one compound and in amounts less than 1%; ethyl oleate has been reported to have significant larvicidal, repellent, ovicidal, and adulticidal properties against *Tetranychus cinnabarinus* [78].

The results of this study are important for future research, as most of the identified compounds do not report their biological activities. Although most of the identified compounds are related to antimalarial activity, it is necessary to perform this activity in a way that it verifies the medicinal use of this plant for treatment, since compounds that are in concentrations above 4%, such as nerolidol and ethyl 5-oxo-4-(p-toluidino)-2,5-dihydro-3-furancarboxylate, are assumed to be the main contributors to this activity.

The results of the compounds identified by GC/MS provided information that allowed relating them to ethnomedicinal use and biological activities. Ethnomedicinal uses such as pain caused by gallstones and kidney stones, toothache, abdominal pain, menstrual cramps, and prostate inflammation were related to analgesic and anti-inflammatory activities, malaria to antimalarial activity, intestinal parasites to antiparasitic, anthelmintic, antibacterial, and/or antimicrobial activity, and ovarian cysts to antioxidant, anticancer, and antitumor activities. It is necessary to isolate the compounds and test their activity individually and/or in combination to see the correlative effect between them.

### 4.4. Antibacterial Activity Assessment

The traditional approach of preparing the extract using water was used, which produced less antibacterial activity than the preparation with the ethanol extract. This could be due to the polarity of the solvents used (water and ethanol), where the solubility of various plant compounds in solvents of different polarities is involved. It is established that the polarity of the solvent affects the qualitative and quantitative compositions of bioactive compounds enriched in various solvents [79], which is reflected in this study, since ethanol is an extractant more powerful than water, thus justifying the high inhibition zones of the ethanol extract.

The bark extract of *M. americana* L. showed the capacity to inhibit the bacterial growth of *Staphylococcus aureus* and *Escherichia coli*, with *S. aureus* having a greater activity in each of the concentrations used, coinciding with other studies conducted on the plant, where the extracts were more active against Gram-positive bacteria than Gram-negative bacteria [21, 22]. The behavior in the inhibition zone for the ethanolic and aqueous extracts was similar, since in both extracts the diameter of the inhibition halos decreased as the concentrations of the extracts decreased.

As the ethanolic extract produced higher inhibition activity than the raw water extract in the worked concentrations, a significant difference was shown against the worked concentrations in each of the extracts, which were the same for each of the bacterial strains, in that sense, as for the *S. aureus* strain, evaluating the concentrations of the ethanolic extract vs. the aqueous extract (ANOVA: 20 mg/ml, mean diff. 4.067, *p*=0.0018; 10 mg/ml, mean diff.: 3.433, *p* < 0.0001; 5 mg/ml, mean diff.: 2.967, *p*=0.0117), and *E. coli* (ANOVA: 20 mg/ml, mean diff.: 7.200, *p* < 0.0001; 10 mg/ml, mean diff.: 6.667, *p*=0.0021; 5 mg/ml, mean diff.: 5.067, *p*=0.0154). These results are in agreement with studies made with the methanolic and aqueous extracts of *A. pluriseta* Schweinf. (Asteraceae) tested against the microbes *S. aureus*, *E. coli*, and *C. albicans* (ANOVA, *S. aureus*, F (3.8) = 160.1, *p* < 0.001; *E. coli*, F (3.8) = 53.67, *p* < 0.001; *C. albicans*, F (3.8) = 72.67, *p* < 0.001), and the extract of raw water showed antimicrobial activity below 7 mm [73].

Although the concentrations of the extracts used were in the range of 10^2^ times more than the antibiotic used as a positive control (streptomycin), the extracts showed remarkable antibacterial activity, as shown by their inhibition zones, highlighting the value of 22.6 ± 0.1 for the case of *S. aureus* and 19.6 ± 0.17 for the case of *E. coli*. The above could be due to the active components of the extracts, which present only a fraction of them, deducing that, if the components are isolated and purified, they could show greater antibacterial activity, represented through their inhibition zone. It is expressed that the concentration of the active components in the extracts could be much lower than the standard antibiotics [72]. In the strains of *B. subtilis* and *P. aeruginosa,* there was no antibacterial activity for any of the plant fractions, which could be related to the low permeability of the cell wall to antibacterial agents [22].

Similarly, Mosquera et al. [21] reported that endophyte fungal extracts of the leaves and seeds of this plant inhibited the growth of *Staphylococcus aureus* and *Escherichia coli* strains by producing inhibition zone diameters ranging 12–22 mm and 12–23 mm, respectively. Similarly, in the work of Rodriguez et al. [22], the cortex was only active against the *S. aureus* strain, with inhibition halos of 17 mm with a concentration of 780 mg/ml.

The antimicrobial activity of the ethanolic extract of *M. americana* showed higher inhibition zones against Gram-positive bacteria, specifically against the bacterial strain *S. aureus*, with inhibition zones (22.6 mm) very close to those demonstrated by the positive control, standard drug (streptomycin: 28.4 mm). Dissanayake et al. [33] indicated that the root bark of *P. indica* has Plumbagin as an active compound, revealing a strong antimicrobial activity in the methanolic extract against Gram-positive bacteria *S. aureus*, with an inhibition zone around 24 mm, a value equivalent or similar to that obtained in the assay with the standard drug chloramphenicol, with an inhibition zone of 28.0 mm. Gram-positive bacteria are more susceptible to antibacterial activity. This resistance of Gram-negative bacteria could be due to the low permeability of the cell wall to bacterial agents, high genetic capacity to express a wide repertoire of resistance mechanisms, by mutations in chromosomal genes that regulate the expression of resistance genes and by the acquisition of foreign resistance genes via plasmids, transposons, and bacteriophages [80].

In addition to the above, previous findings of another species of the *Mammea* genus, *Mammea suriga*, exhibited strong antibacterial activity in aqueous and ethanolic extracts showing zones of inhibition between 32 and 33 mm against *S. aureus*; on the other hand, a zone of inhibition between 20 and 25 mm was presented against *B. subtilis*; as for the bacterial strain *E. coli*, a zone of moderate inhibition was registered in all the extracts in which they were evaluated [45].

The results show that this antibacterial activity could be related to the secondary metabolites found in this study, such as tannins, alkaloids, flavonoids, terpenes, and quinones [7, 47, 48, 81, 82]. The antibacterial activity could be justified by the presence of the compounds identified in this study. Compounds, such as nerolidol, *δ*-cadinene, caryophyllene, calarene, caryophylladienol II, epizonarene, and *γ*-gurjunene, present this activity against *S. aureus* and *E. coli* in several groups of plants, which may be due to the synergistic effect of the components found [49, 83–88].

### 4.5. Minimum Inhibitory Concentration (MIC) of the Crude Extracts

MIC is considered a fundamental parameter for testing the sensitivity of a bacterium to an antibacterial [89]. The minimum inhibitory concentration performed in this study showed that aqueous and ethanolic extracts of *M. americana* exhibited strong activity against *S. aureus* and *E. coli* strains. The MIC of the extracts ranged from 0.0503 to 0.191 mg/ml. Among the two bacterial strains tested, *S. aureus* proved to be more sensitive, with a MIC of 0.0503 mg/ml for the ethanolic extract. According to Avellana et al., a bacterial strain is very sensitive when the substance tested has a MIC below 12.5 mg/ml, medium sensitivity between 12.5 and 50 mg/ml, and low sensitivity for values between 50 and 100 mg/ml [90]. In this order of ideas, the results obtained in this study indicate a high bacterial sensitivity in the extracts evaluated, since the MIC values were below 12.5 mg/ml, thus determining the antibacterial potential of the species. However, Kuete states that the activity of crude extracts is significant if MIC values are below 100 *μ*g/ml, moderate when 100 < MIC < 625 *μ*g/ml, or low when MIC > 625 *μ*g/ml [91], which would indicate that in this study, the bacterial activity would be between significant and moderate, in the ethanol and aqueous extracts, respectively.

Few articles have reported the antibacterial potential of *M. americana*. Mosquera et al. demonstrated that the endophytic fungi found in the leaves and seeds of this species possess bactericidal characteristics against *S. aureus* and *E. coli*, with MIC ranging from 1.0 *μ*l/ml to 2.0 *μ*l/ml [21]. Bactericidal activity is reported in fruit peel extracts with hexane, ethyl acetate, and acetone and seed extracts in methanol against *S. aureus*, with MIC of 8, 16, 4, and 2 *μ*g/ml, respectively [52, 92, 93].

Among studies conducted in other *Mammea* species (*M. africana*), the extract of this plant and its active component *Mammea* A/AA did not inhibit the growth of *E. coli* but did inhibit the growth of other bacteria such as *Streptococcus pneumonia*, *Clostridium difficile*, and *Campylobacter jejun*i, with MIC ≤ 2 *μ*g/ml [94]. The presence of lipopolysaccharides in the outer membrane of Gram-negative bacteria prevents the presence of hydrophobic antimicrobial agents, so they are less susceptible to extracts or essential oils containing hydrophobic antimicrobial compounds [95].

## 5. Conclusions

This research presents different ethnomedical uses of the bark of *M. americana* L. in the municipality of Cértegui. Diseases or symptoms such as gallstones, prostate inflammation and pain, malaria, and intestinal parasites are the most representative.

The phytochemical analysis carried out confirmed the existence of different secondary metabolites in its ethanolic extract, such as tannins, alkaloids, flavonoids, triterpenes and/or steroids, quinones, saponins, and sapogenins. In addition, 29 compounds were identified and quantified. The information given by the phytochemical analysis followed by its identification and quantification served to associate these results with the medicinal uses given by the community under study; thus, the analgesic and anti-inflammatory activities were related to diseases such as pain caused by kidney stones, gallstones, toothache, menstrual cramps, inflammation and pain in the prostate, antimalarial activity of malaria, and antitumor activity of ovarian cysts.

The antibacterial activity carried out on the extract of the bark of *Mammea americana* showed a significant effect against the bacterial strains *Staphylococcus aureus* and *Escherichia coli.* The presence of identified compounds in this plant, such as nerolidol, *δ*-cadinene, caryophyllene, calarene, caryophylladienol II, epizonarene, and *γ*-gurjunene, could scientifically justify this activity; however, work is required such as the isolation and purification of the compounds, in such a way that it is possible to decipher the mechanism of action.

Further studies are needed to validate the industrial applications of *M. americana* L. for medicinal use. It is recommended to carry out more research studies on the pharmacological properties of this species in vivo, which allows validating the different activities it presents according to the medicinal reports given by the community, which allows associating the disease with antimalarial, analgesic, anti-inflammatory, and antimicrobial activities.

## Figures and Tables

**Table 1:** Summary of ethnomedicinal information and demographic characteristics of the people surveyed (*n* = 110).

Parameters	Group	Values	Percentage (%)
Gender	Masculine	37	33.6
	Female	73	66.4
Years of age	21–30	9	8.2
	31–40	19	17.3
	41–50	22	20.0
	51–60	32	29.1
	61–70	21	19.1
	71–80	7	6.4
Occupation	Mining	41	37.3
	Woodworking	29	26.4
	Housewife	23	20.9
	Farmer	5	4.5
	Teacher	3	2.7
	Student	2	1.8
	Driver	2	1.8
	Healer	2	1.8
	Speaker	1	0.9
	Merchant	1	0.9
	Various jobs	1	0.9
Diseases/symptoms	Gallstones	49	44.5
	Prostate inflammation	12	10.9
	Malaria	11	10.0
	Intestinal parasites	9	8.2
	Anemia	8	7.3
	Laxative (to treat constipation)	5	4.5
	Toothache	4	3.6
	Hypercholesterolemia	3	2.7
	Menstrual cramps	3	2.7
	Hepatitis and fatty liver treatment	3	2.7
	Abdominal pain (stomach pain)	1	0.9
	Ovarian cysts	1	0.9
	Kidney stones	1	0.9

**Table 2:** Results of physicochemical analysis of extract from the bark of *M. americana* L.

Qualitative tests (metabolites)	Ethanolic extract
Tannins	
Ferric chloride test	+
Test with lead acetate	+
Test with gelatine-salt	+
Alkaloids	
Mayer test	++
Flavonoids	
Shinoda test	+
Hydrochloric acid test	+
Triterpenes and/or steroids	
Liebermann–Burchard	+++
Quinones	++
Coumarins	
Fluorescence reaction	−
Ferric hydroxamate reaction	−
Ehrlich's reaction	−
Saponins and sapogenins	
Foam reaction	+

(+++), high presence; (++), medium presence; (+), low presence; (−), absence.

**Table 3:** Components identified by GC/MS in ethanolic extract from the bark of *M. americana* L.

*N*	Compound	Retention time	Relative amount (%)	Experimental molecular mass (g/mol)	Theoretical molecular mass (g/mol)
Sesquiterpenes			4.3	204	204.35
1	*δ*-Elemene	8.829	0.09	204	204.35
2	*α*-Cubebene	9.629	0.26	204	204.35
3	Germacrene A	9.955	0.27	204	204.35
4	Caryophyllene	10.583	0.16	204	204.35
5	Calarene	10.824	0.11	204	204.35
6	Caryophylladienol II	11.03	0.13	204	204.35
7	(E)-*β*-farnesene	11.219	0.19	204	204.35
8	Epizonarene	11.829	0.5	204	204.35
9	*α*-Elemene	12.19	1.38	204	204.35
10	*δ*-Cadinene	12.723	1.13	204	204.35
11	*γ*-Gurjunene	14.296	0.08	204	204.35
Sesquiterpenes oxygenates			4.92		
12	Nerolidol	13.66	4.49	204	204.35
13	1-Formyl-2,2-dimethyl-3-trans-(3-methyl-but-2-enyl)-6-methylidene-cyclohexane	14.073	0.13	220	220.35
14	Dihydro-cis-*α*-copaene-8-ol	15.01	0.26	222	222.3663
15	2-Propenoic acid, 3-[4-[(3-methyl-1-butenyl) oxy] phenyl]-, methyl ester	24.903	0.04	246	246.3
Triterpenes			0.21		
16	Squalene	43.985	0.21	410	410.391
Monoterpenes			0.05		
17	3,7-Dimethyl-oct-6-enoic acid, ethyl ester	27.817	0.05	198	198.3
Diterperne			4.28		
18	19-Hydroxy-13-epimanoyl oxide	26.382	4.28	306	306.48
Phenylpropanoid			0.26		
19	2-Chromancarboxylic acid, 6-amino-4-oxo-, ethyl ester	13.351	0.26	235	235.236
Alkaloid			75.96		
20	Ethyl 5-oxo-4-(p-toluidino)-2,5-dihydro-3-furancarboxylate	22.548	73.7	261	261.3
21	Pyrido[2,3-b]pyrimido[4,5-d]thiophen-4 (3H)-one, 3-amino-9-methoxymethyl-2,7-dimethyl	23.769	0.99	275	275.26
22	5,6,7-Trimethoxy-2,3-dihydrofuro (2,3-b) quinoline	24.422	0.05	261	261.27
23	Atherospermidine	28.032	0.05	305	305.28
24	4,5,6,7-Tetrahydro-benzo[c]thiophene-1-carboxylic acid allylamide	28.995	0.06	221	221.319
25	9-Phenylcarbazole	29.82	0.41	243	243.3
26	1-(1-Benzothien-2-yl)-N, N-diethylcyclohexanamine	34.762	0.65	287	287.463
27	Methyl-methoxy-(1,1-difluoro-2,2-bis-trifluoromethyl-ethyl) amine	53.449	0.05	261	261.11
Phenolic compound			9.83		
28	Phenol, 4,4′,4″-ethylidynetris	26.837	9.83	306	306.35
Unsaturated fatty acid			0.21		
29	Ethyl oleate	27.19	0.21	310	310.5
Total			100%		

**Table 4:** Results of antibacterial activity of *M. americana* bark extract (*n* = 3).

Strain/extract	Ethanolic extract (mg/ml)					Aqueous extract (mg/ml)					Positive control (streptomycin) (10 *μ*g/ml)	Negative control
	20	10	5	2.5	0.5	20	10	5	2.5	0.5
	The diameter of zone of inhibition												
*Bacillus subtilis* (ATCC 6633)	—	—	—	—	—	—	—	—	—	—	24.5 ± 0.15	—
*Staphylococcus aureus* (ATCC 25923)	22.6 ± 0.10	18.5 ± 0.10	15.6 ± 0.12	10.2 ± 0.15	8.2 ± 0.25	18.5 ± 0.15	15.1 ± 0.15	12.6 ± 0.25	6.4 ± 0.10	2.8 ± 0.12	28.4 ± 0.21	—
*Escherichia coli* (ATCC 25922)	19.6 ± 0.17	16.8 ± 0.25	13.4 ± 0.44	6.4 ± 0.15	3.3 ± 0.12	12.4 ± 0.10	10.1 ± 0.10	8.3 ± 0.15	3.4 ± 0.15	1.5 ± 0.15	28.4 ± 0.21	—
*Pseudomonas aeruginosa* (ATCC 13076)	—	—	—	—	—	—	—	—	—	—	20.4 ± 0.21	—

**Table 5:** Minimum inhibitory concentration (MIC) of aqueous and ethanol extracts of *M. americana.*

Bacterial strains	Aqueous extract (mg/ml)	Ethanolic extract (mg/ml)
*S. aureus*	0.116 ± 0.058	0.0503 ± 0.0021
*E. coli*	0.191 ± 0.004	0.074 ± 0.0015

## Data Availability

The data used to support the findings of this study are included within the supplementary information file.
