# Supplementary material for: Ethnomedicinal Studies, Chemical Composition, and Antibacterial Activity of the Mammea americana L. Bark in the Municipality of Cértegui, Chocó, Colombia
Source: Adv Pharmacol Pharm Sci. 2022 Jan 19;2022:9950625. doi: 10.1155/2022/9950625 (PMC8791731; doi:10.1155/2022/9950625)
Supplement: Supplementary Materials — The following are available online. Figure S1. Fragmentation patterns of the mass spectra of the secondary metabolites present in the ethanolic extract of M. americana bark. [file 9950625.f1.zip › 9950625.f1/Figure S1_10032021.pdf]

δ-Elemene

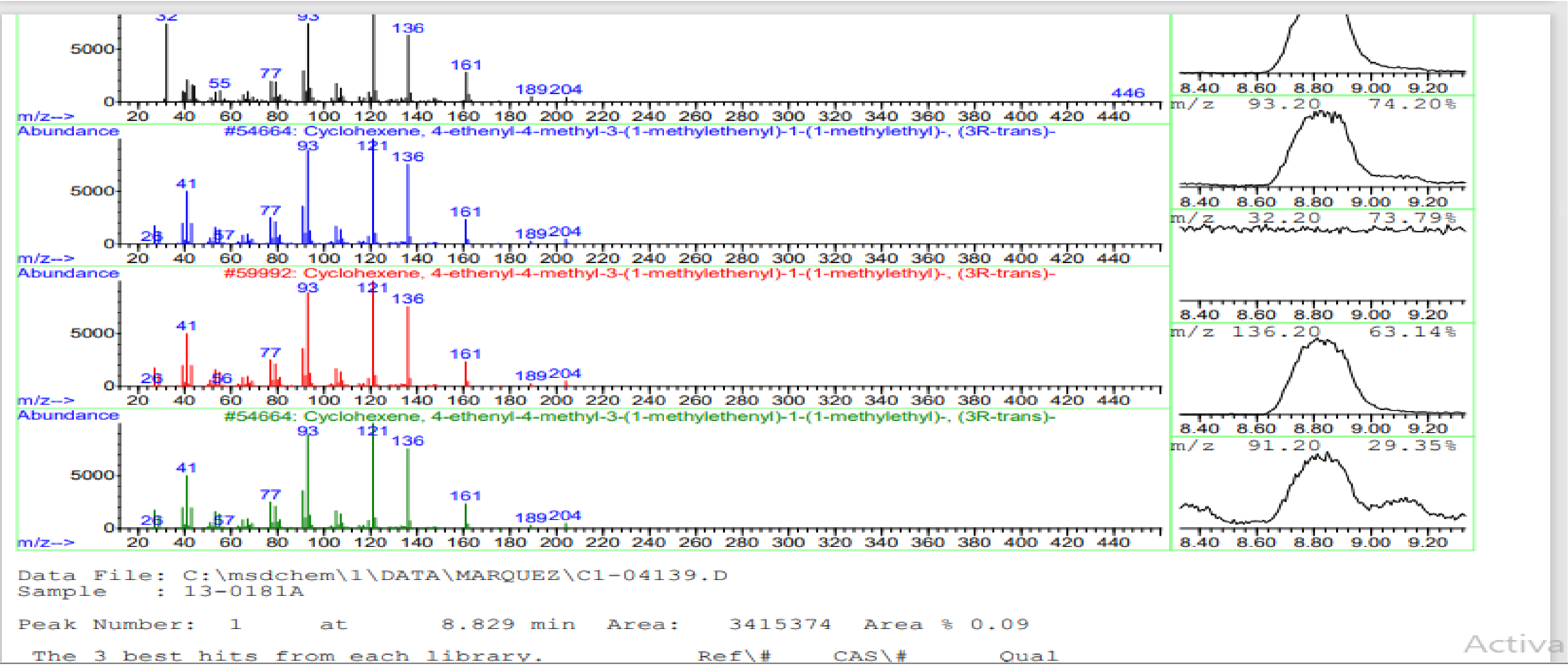

#54664: Cyclohexene, 4-ethenyl-4-methyl-3-(1-methylethenyl)-1-(1-methylethyl)-, (3R-trans)-
#59992: Cyclohexene, 4-ethenyl-4-methyl-3-(1-methylethenyl)-1-(1-methylethyl)-, (3R-trans)-
#54664: Cyclohexene, 4-ethenyl-4-methyl-3-(1-methylethenyl)-1-(1-methylethyl)-, (3R-trans)-
Abundance
m/z-->
m/z 93.20 74.20%
m/z 32.20 73.79%
m/z 136.20 63.14%
m/z 91.20 29.35%
Data File: C:\msdchem\1\DATA\MARQUEZ\C1-04139.D
Sample : 13-0181A
Peak Number: 1 at 8.829 min Area: 3415374 Area % 0.09
The 3 best hits from each library. Ref\# CAS\# Qual

***α*-Cubebene**

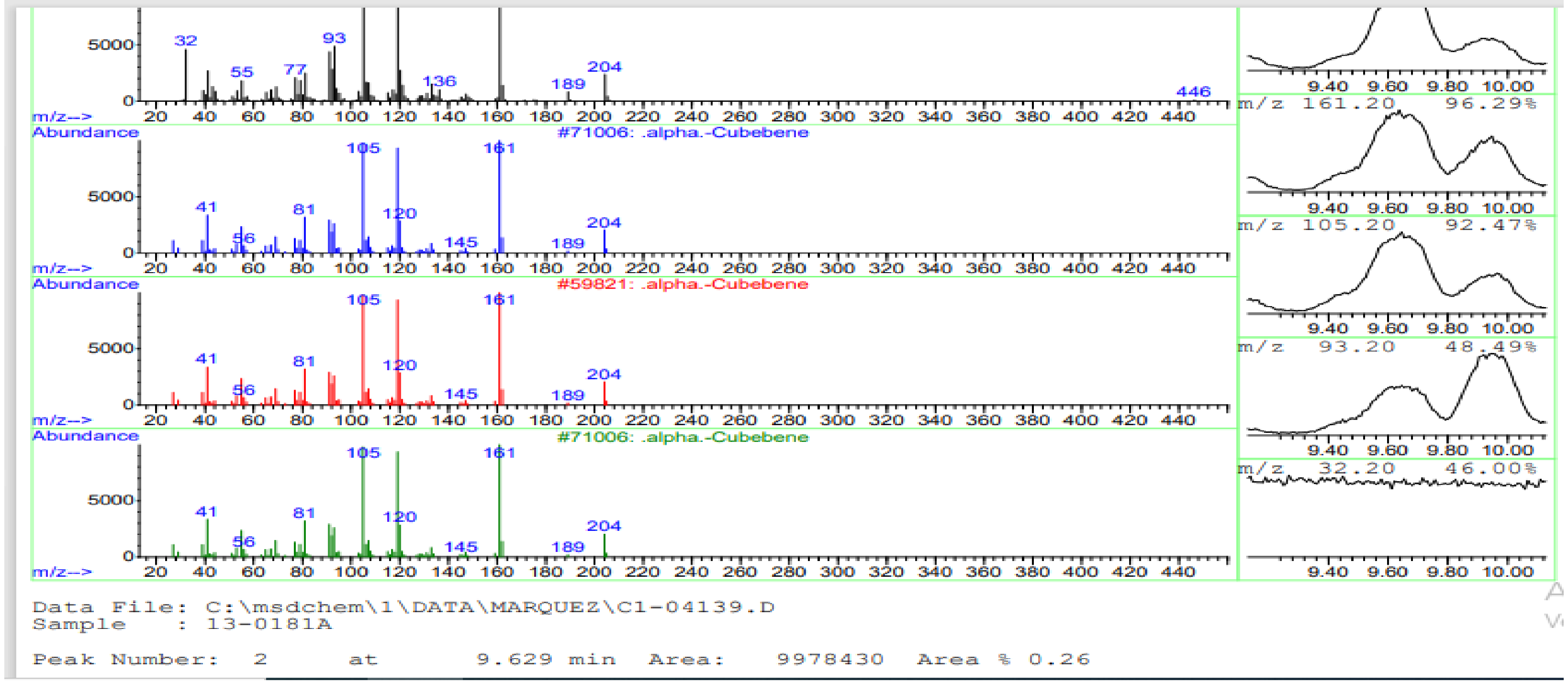

Abundance
#71006: .alpha.-Cubebene
#59821: .alpha.-Cubebene
#71006: .alpha.-Cubebene
m/z-->
m/z 161.20 96.29%
m/z 105.20 92.47%
m/z 93.20 48.49%
m/z 32.20 46.00%
Data File: C:\msdchem\1\DATA\MARQUEZ\C1-04139.D
Sample : 13-0181A
Peak Number: 2 at 9.629 min Area: 9978430 Area % 0.26

Germacreno A

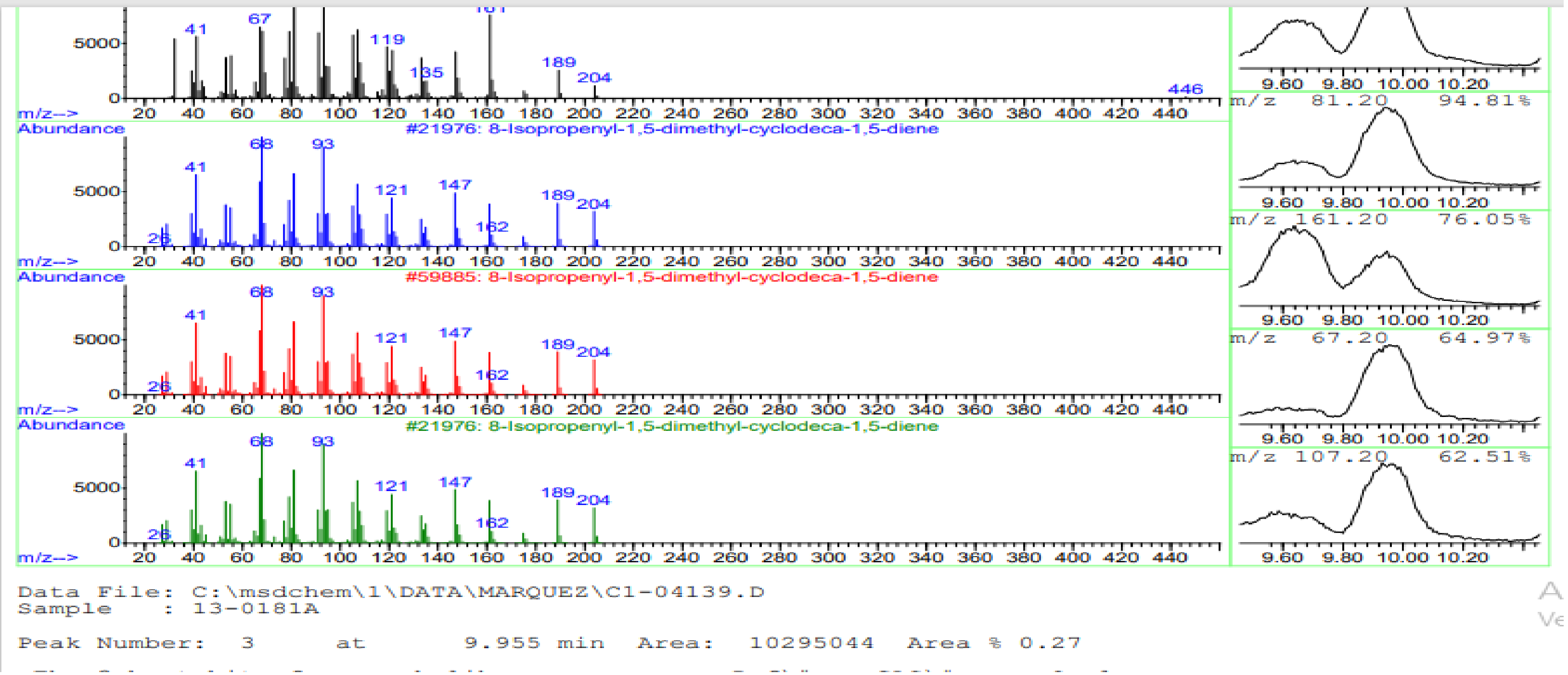

Caryophyllene

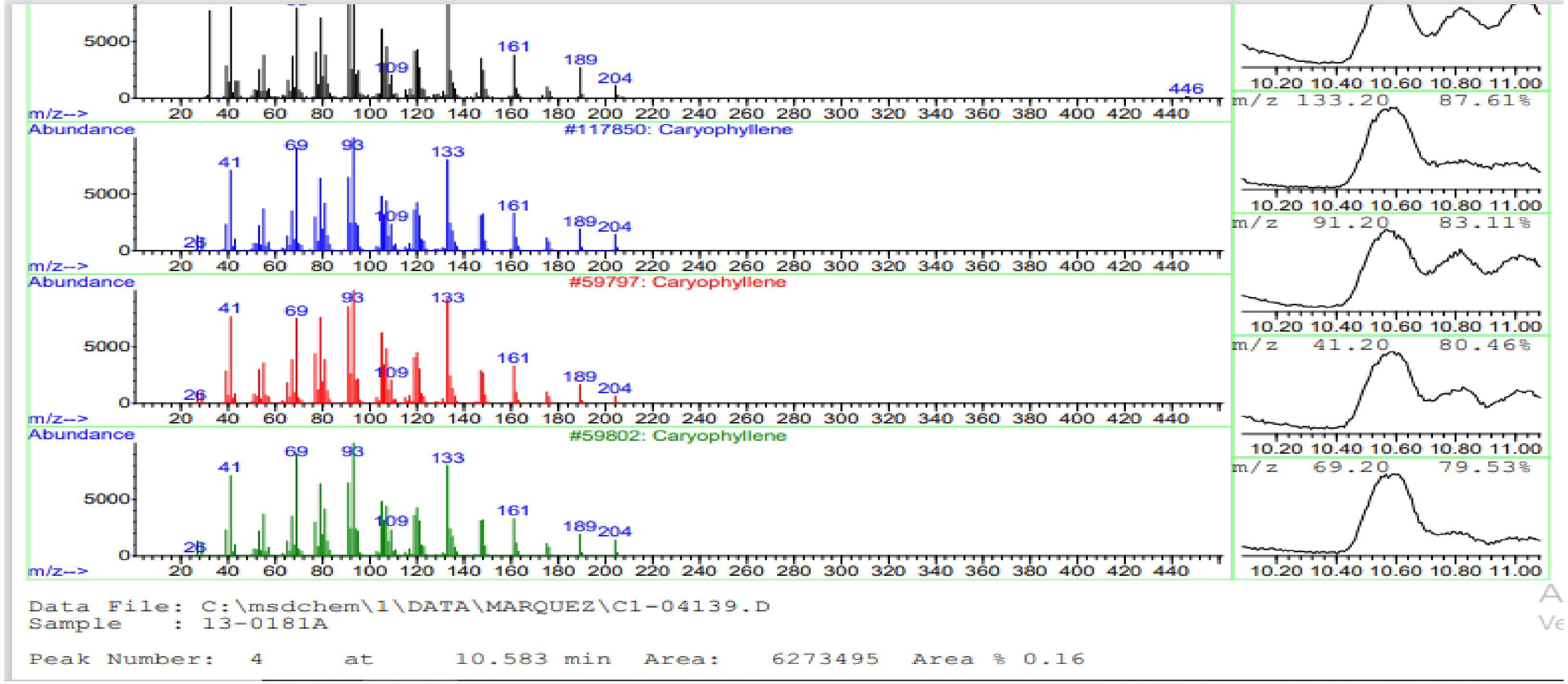

Calarene

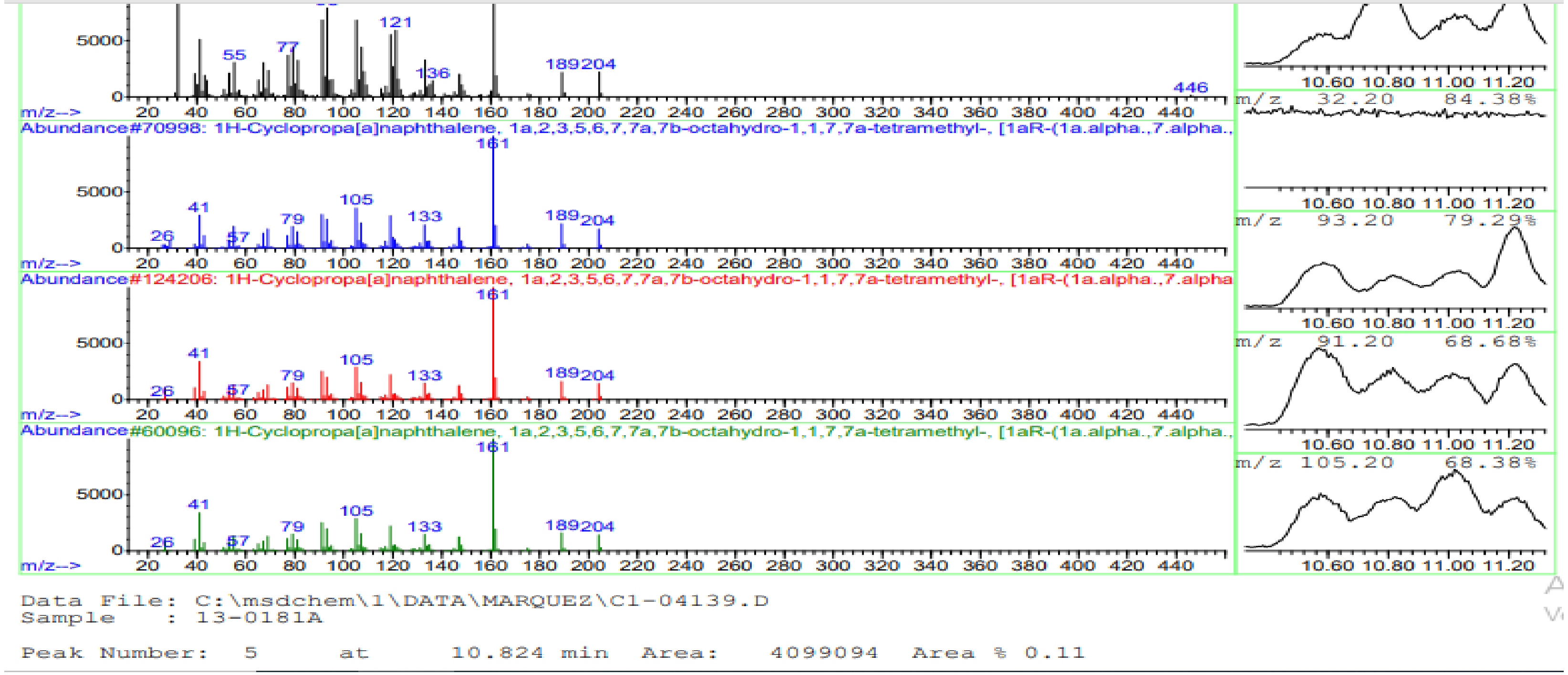
Abundance#70998: 1H-Cyclopropa[a]naphthalene, 1a,2,3,5,6,7,7a,7b-octahydro-1,1,7,7a-tetramethyl-, [1aR-(1a.alpha.,7.alpha.,
Abundance#124206: 1H-Cyclopropa[a]naphthalene, 1a,2,3,5,6,7,7a,7b-octahydro-1,1,7,7a-tetramethyl-, [1aR-(1a.alpha.,7.alpha
Abundance#60096: 1H-Cyclopropa[a]naphthalene, 1a,2,3,5,6,7,7a,7b-octahydro-1,1,7,7a-tetramethyl-, [1aR-(1a.alpha.,7.alpha.,
m/z-->
m/z 32.20 84.38%
m/z 93.20 79.29%
m/z 91.20 68.68%
m/z 105.20 68.38%
10.60 10.80 11.00 11.20
Data File: C:\msdchem\1\DATA\MARQUEZ\C1-04139.D
Sample : 13-0181A
Peak Number: 5 at 10.824 min Area: 4099094 Area % 0.11

Caryophylladienol II

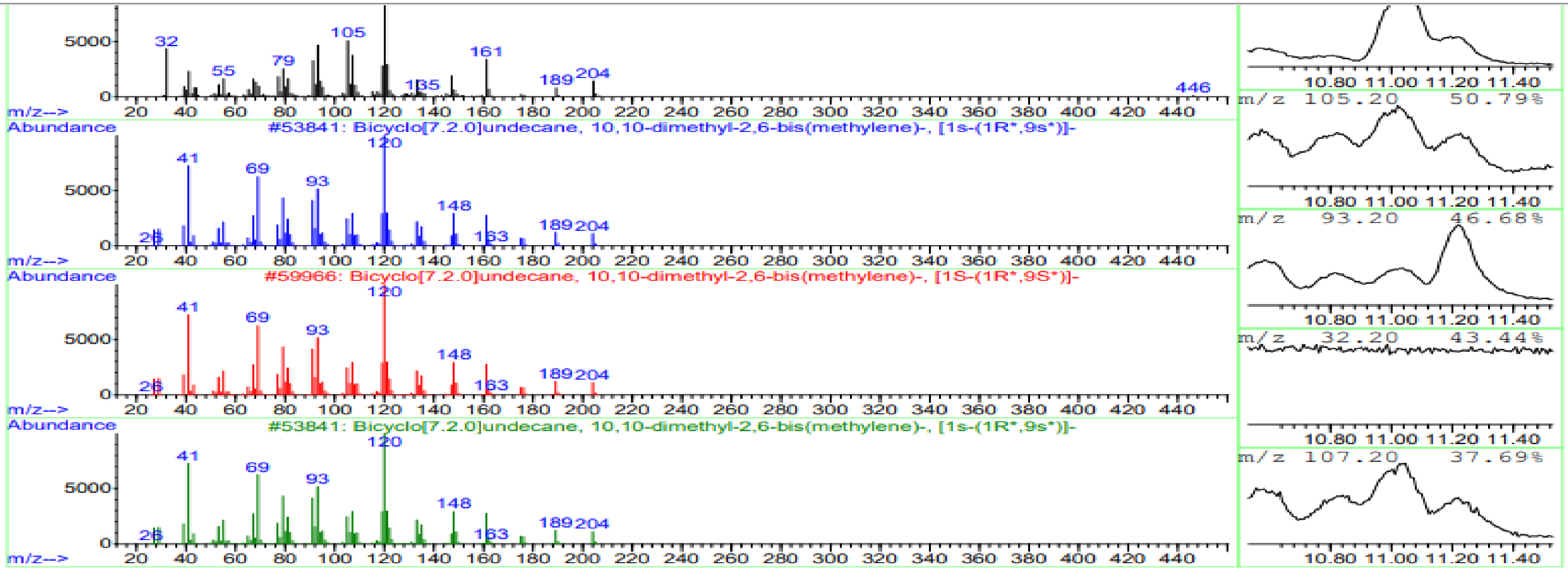

Data File: C:\msdchem\1\DATA\MARQUEZ\C1-04139.D
Sample : 13-0181A

Peak Number: 6 at 11.030 min Area: 5069042 Area % 0.13

(E)-β-Farnesene

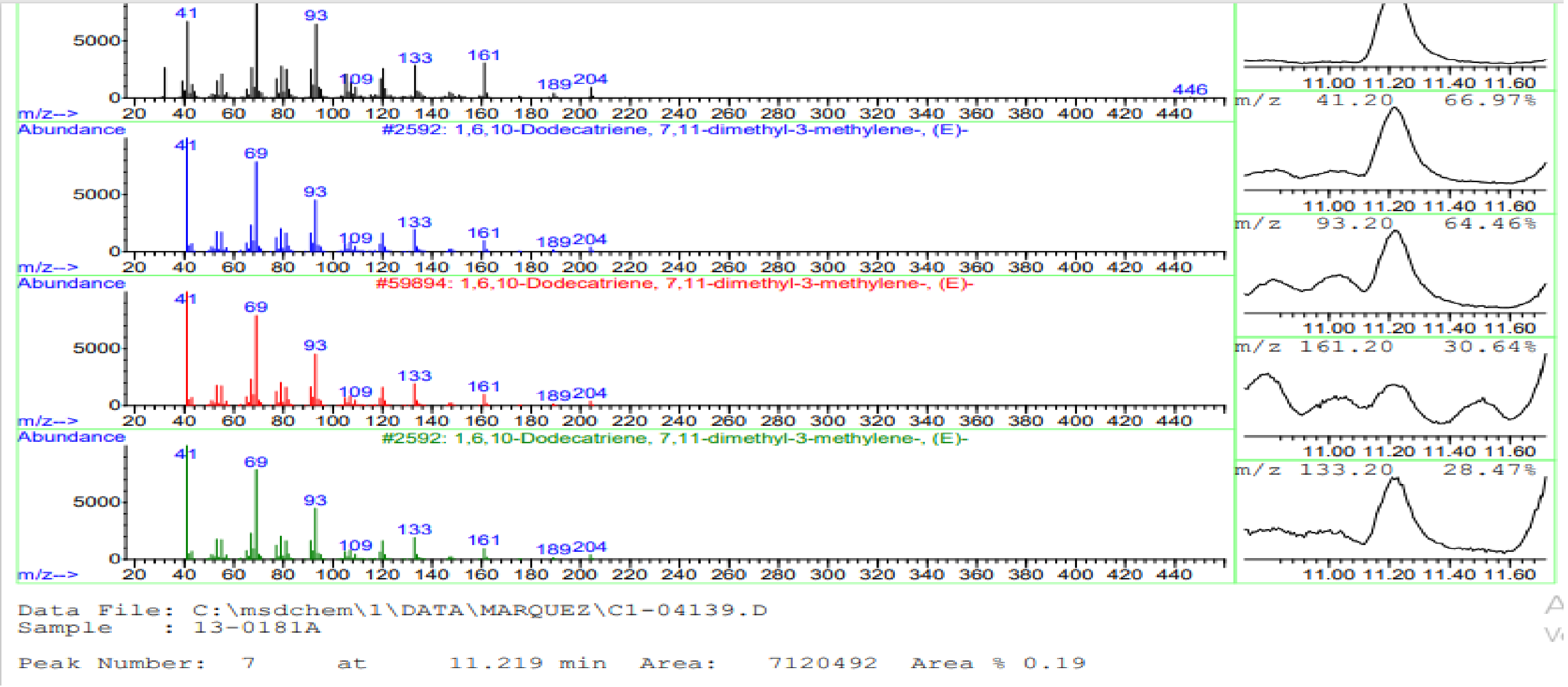

Epizonarene

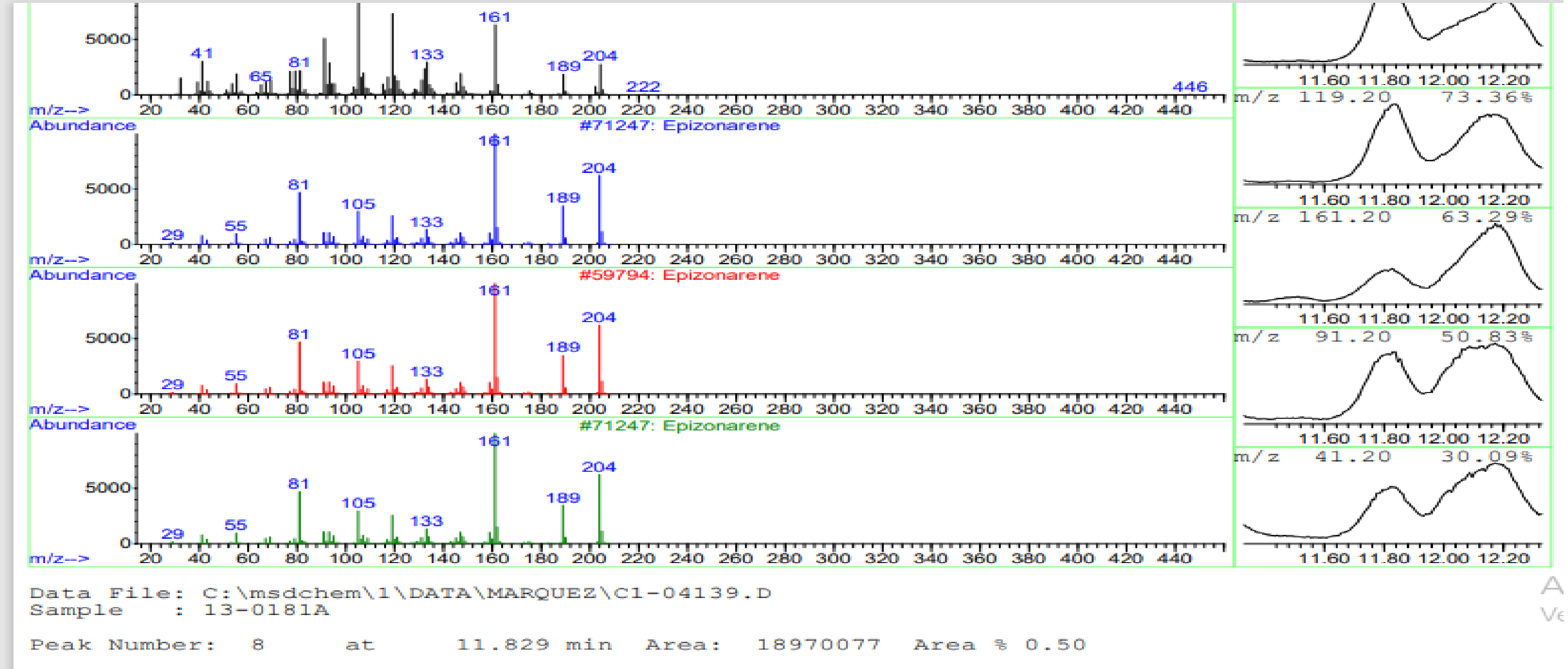

α-Elemene

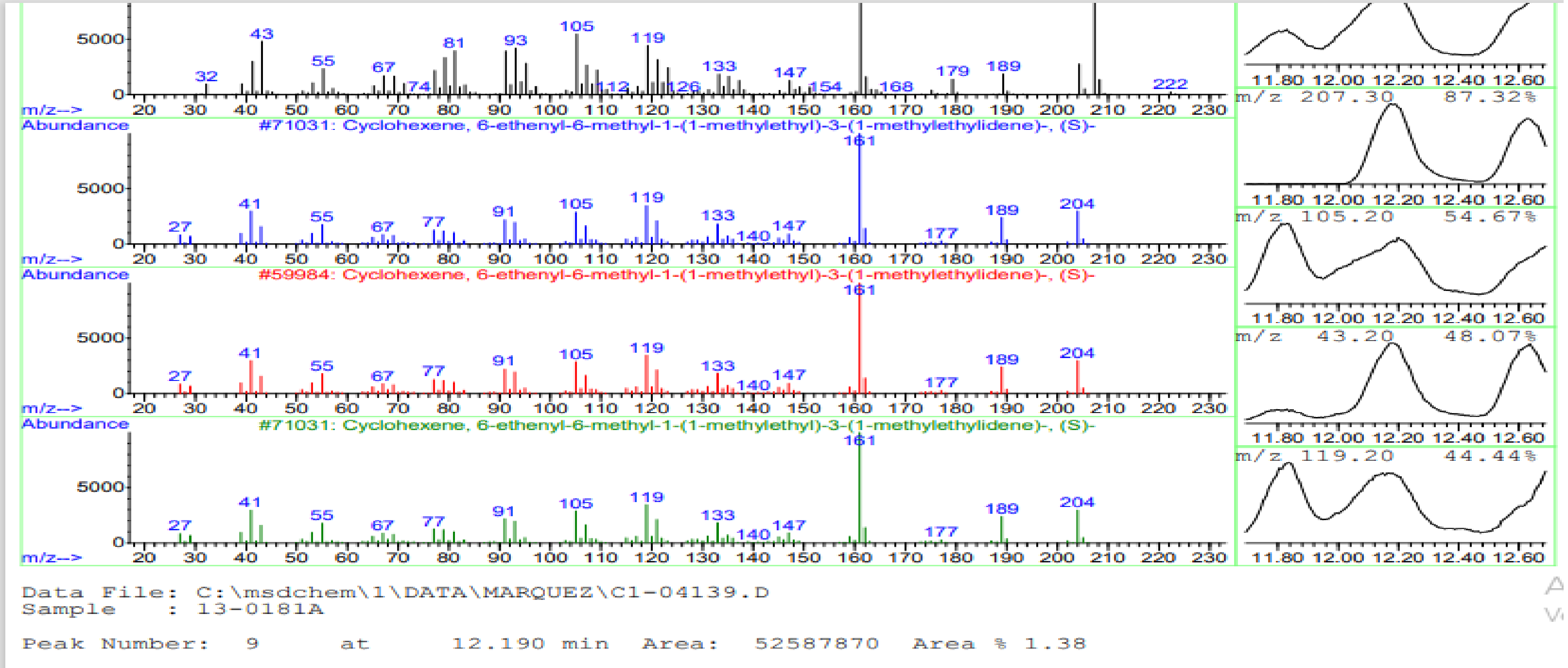
Abundance
#71031: Cyclohexene, 6-ethenyl-6-methyl-1-(1-methylethyl)-3-(1-methylethylidene)-, (S)-
#59984: Cyclohexene, 6-ethenyl-6-methyl-1-(1-methylethyl)-3-(1-methylethylidene)-, (S)-
#71031: Cyclohexene, 6-ethenyl-6-methyl-1-(1-methylethyl)-3-(1-methylethylidene)-, (S)-
m/z-->
m/z 207.30 87.32%
m/z 105.20 54.67%
m/z 43.20 48.07%
m/z 119.20 44.44%
Data File: C:\msdchem\1\DATA\MARQUEZ\C1-04139.D
Sample : 13-0181A
Peak Number: 9 at 12.190 min Area: 52587870 Area % 1.38

δ-Cadinene

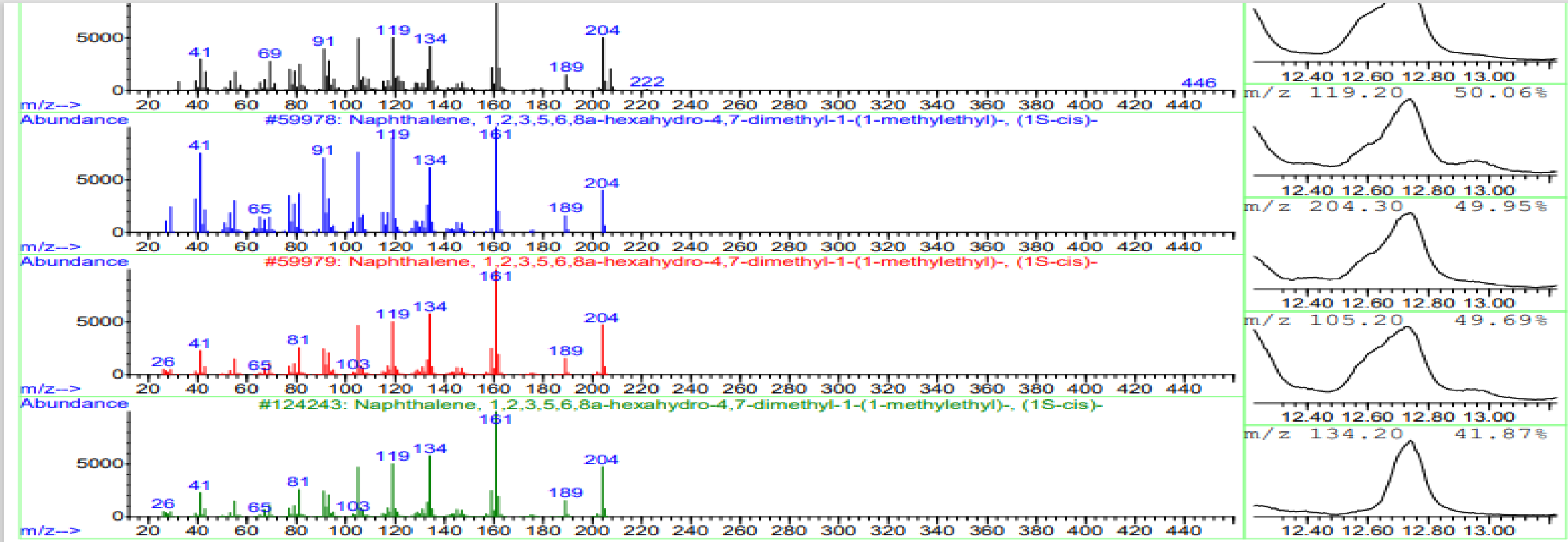

Data File: C:\msdchem\1\DATA\MARQUEZ\C1-04139.D
Sample : 13-0181A

Peak Number: 10 at 12.723 min Area: 43166744 Area % 1.13

γ-Gurjunene

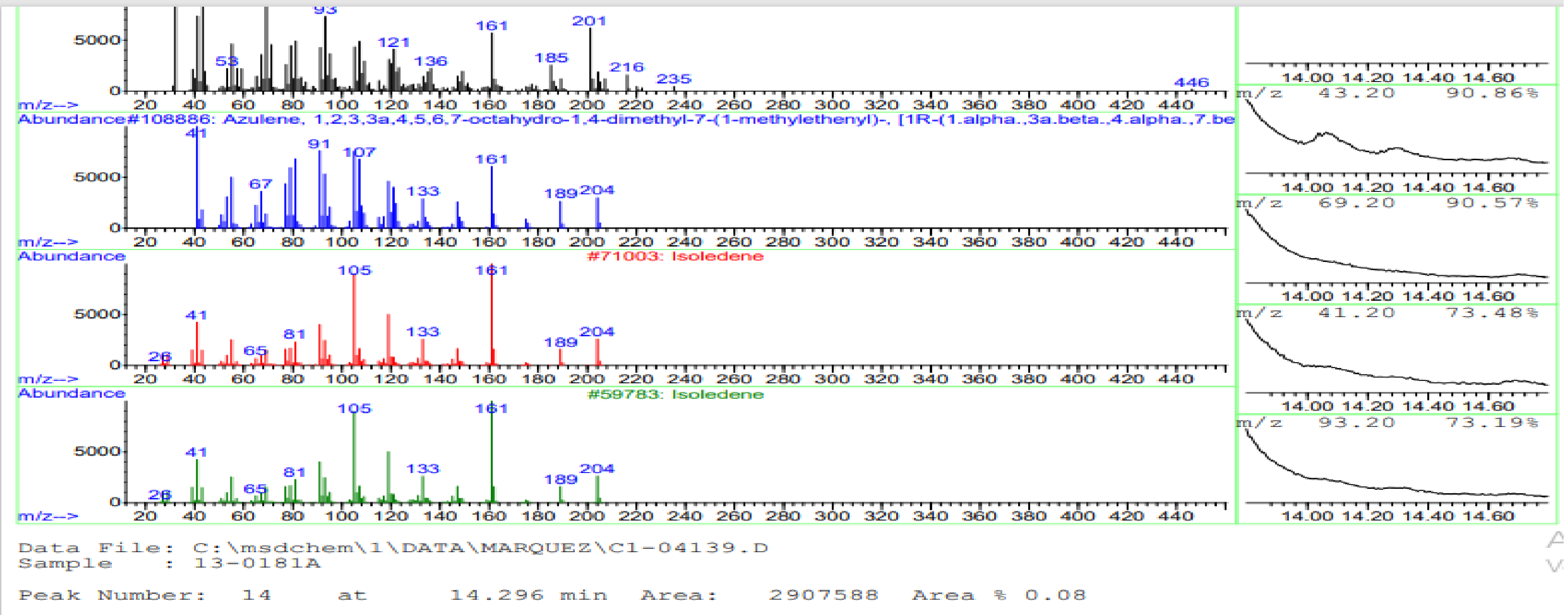
Abundance#108886: Azulene, 1,2,3,3a,4,5,6,7-octahydro-1,4-dimethyl-7-(1-methylethenyl)-, [1R-(1.alpha.,3a.beta.,4.alpha.,7.be
Abundance
#71003: Isoledene
Abundance
#59783: Isoledene
m/z-->
m/z 43.20 90.86%
m/z 69.20 90.57%
m/z 41.20 73.48%
m/z 93.20 73.19%
14.00 14.20 14.40 14.60
Data File: C:\msdchem\1\DATA\MARQUEZ\C1-04139.D
Sample : 13-0181A
Peak Number: 14 at 14.296 min Area: 2907588 Area % 0.08

**Nerolidol**	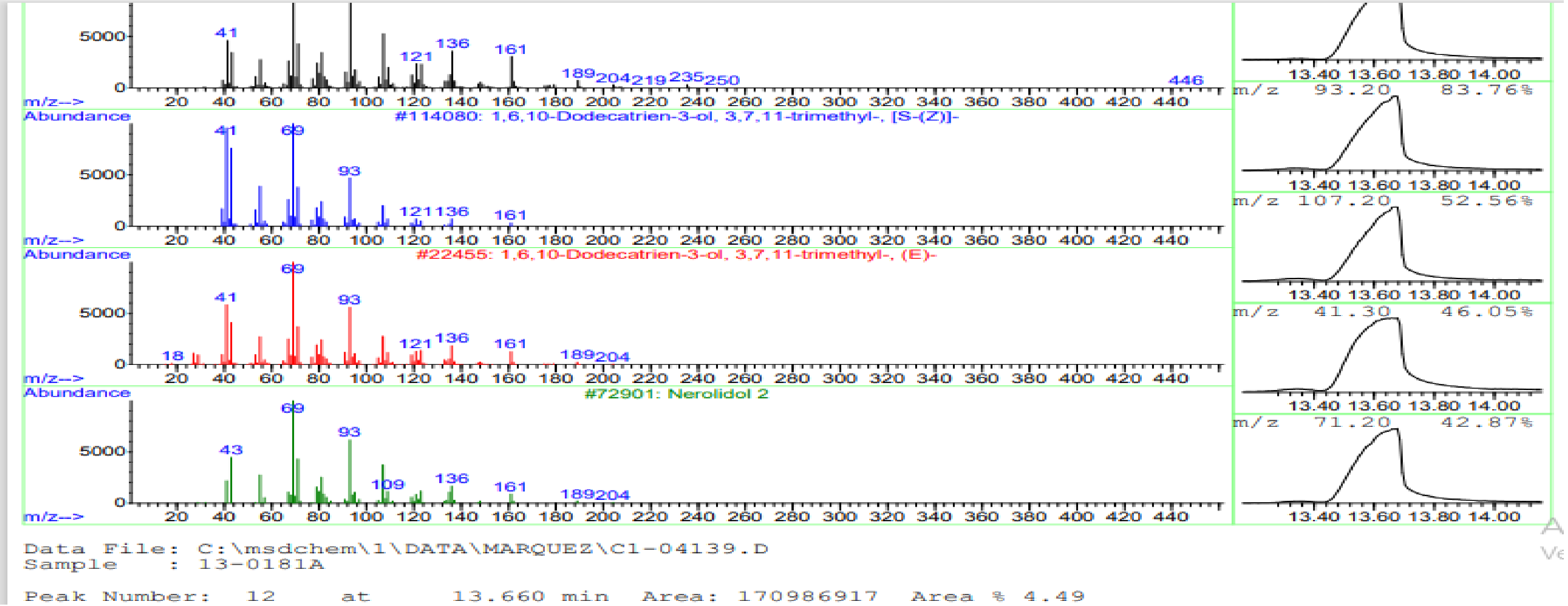

#114080: 1,6,10-Dodecatrien-3-ol, 3,7,11-trimethyl-, [S-(Z)]-
#22455: 1,6,10-Dodecatrien-3-ol, 3,7,11-trimethyl-, (E)-
#72901: Nerolidol 2
Abundance
m/z-->
m/z 93.20 83.76%
m/z 107.20 52.56%
m/z 41.30 46.05%
m/z 71.20 42.87%
Data File: C:\msdchem\1\DATA\MARQUEZ\C1-04139.D
Sample : 13-0181A
Peak Number: 12 at 13.660 min Area: 170986917 Area % 4.49

1-Formyl-2,2-dimethyl-3-trans-(3-methyl-but-2-enyl)-6-methylidene-cyclohexanee

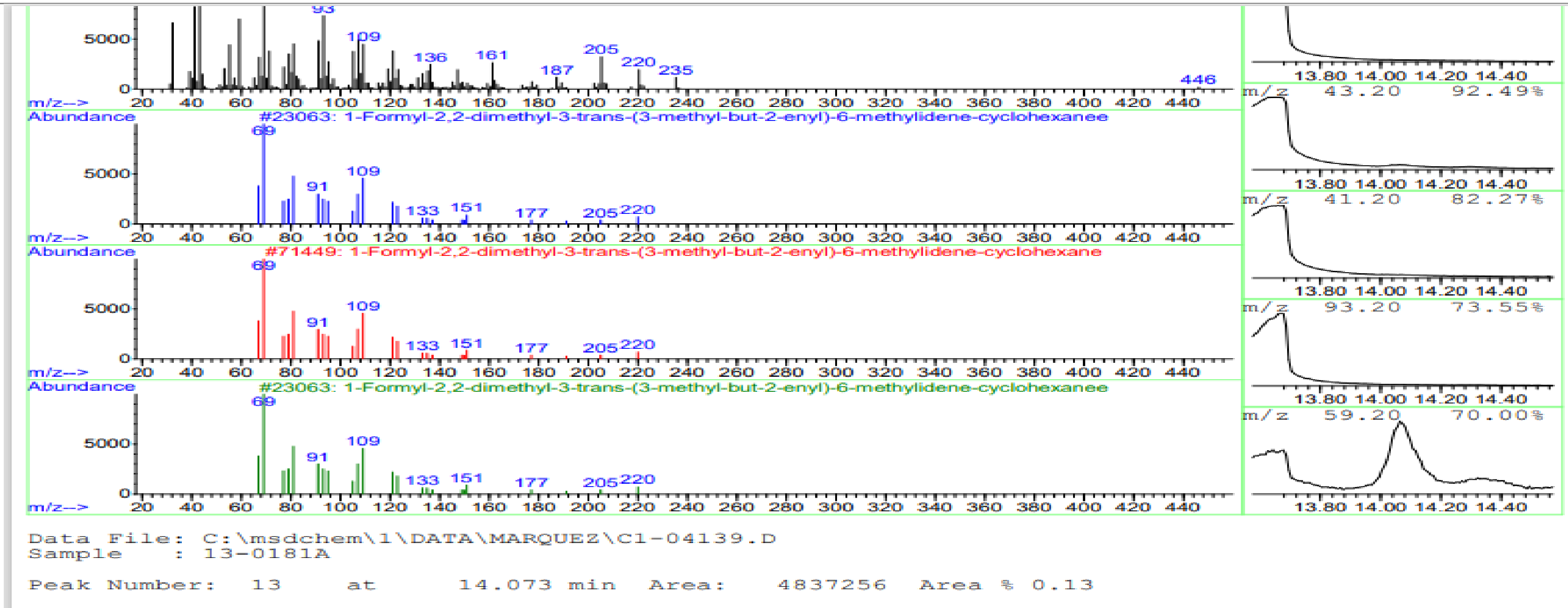

Dihydro-cis-α-copaene-8-ol

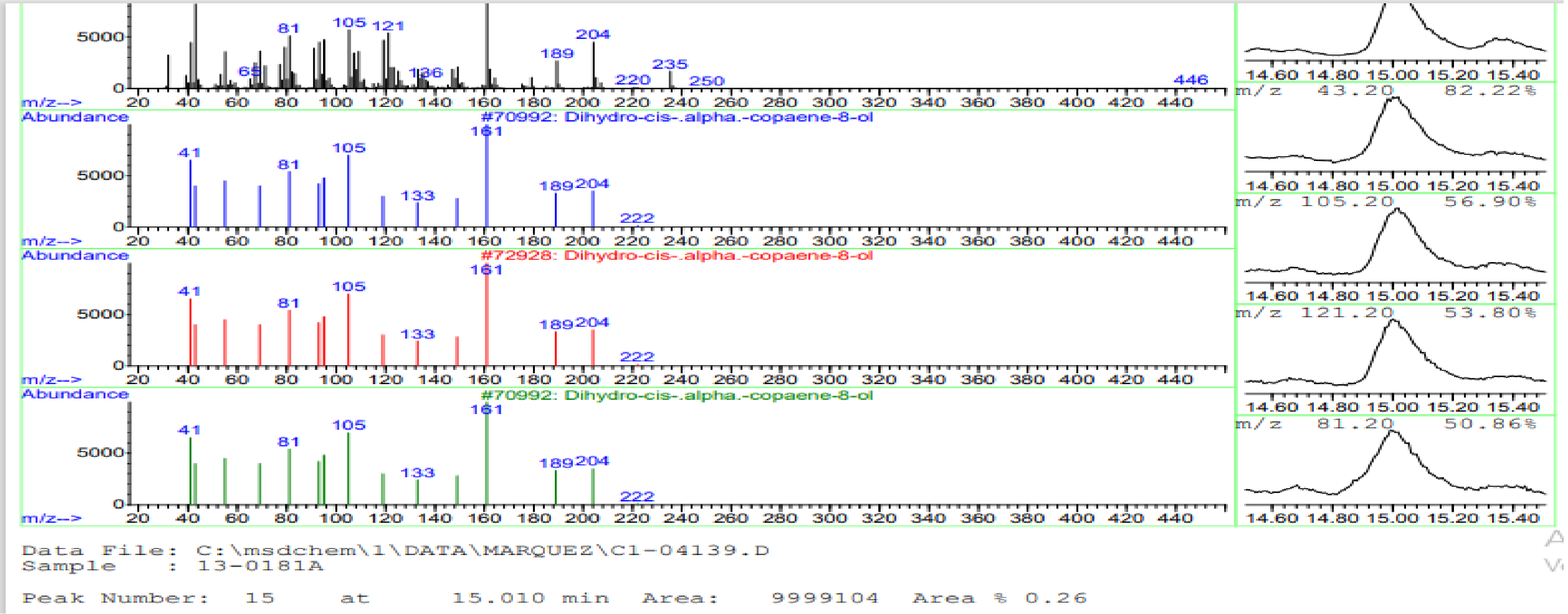

**2-Propenoic acid, 3-[4-[(3-methyl-1-butenyl) oxy]phenyl]-, methyl ester**	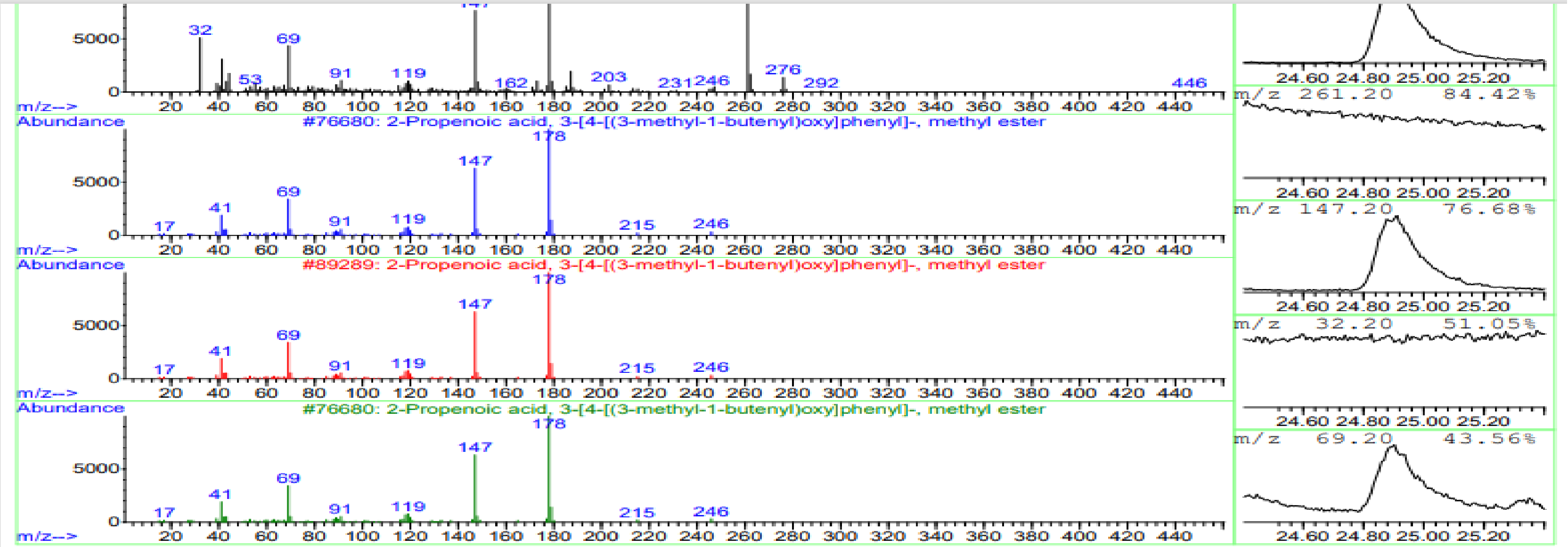<br>Data File: C:\msdchem\1\DATA\MARQUEZ\C1-04139.D<br>Sample : 13-0181A<br>Peak Number: 19 at 24.903 min Area: 1340032 Area % 0.04

Squalene

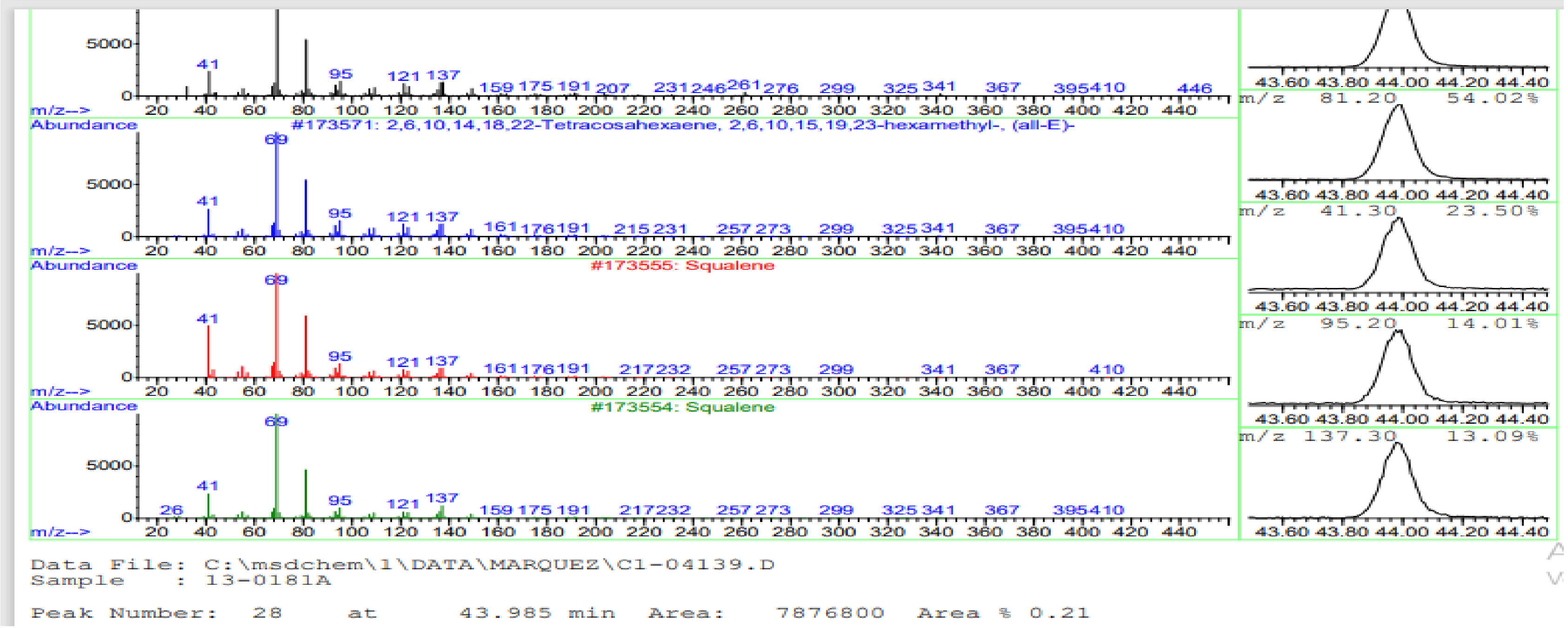

Abundance
#173571: 2,6,10,14,18,22-Tetracosahexaene, 2,6,10,15,19,23-hexamethyl-, (all-E)-
#173555: Squalene
#173554: Squalene
m/z-->
m/z 81.20 54.02%
m/z 41.30 23.50%
m/z 95.20 14.01%
m/z 137.30 13.09%
Data File: C:\msdchem\1\DATA\MARQUEZ\C1-04139.D
Sample : 13-0181A
Peak Number: 28 at 43.985 min Area: 7876800 Area % 0.21

3,7-Dimethyl-oct-6-enoic acid, ethyl ester

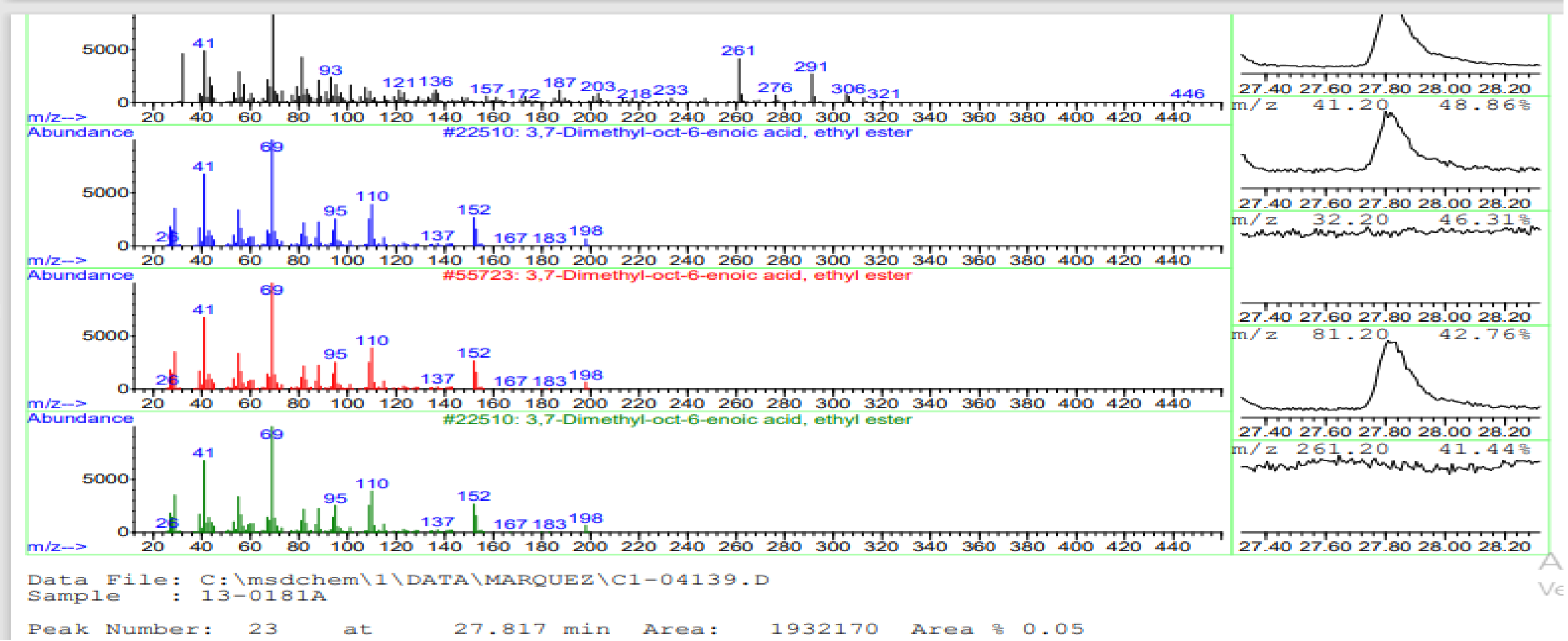

19-Hydroxy-13-epimanoyl oxide

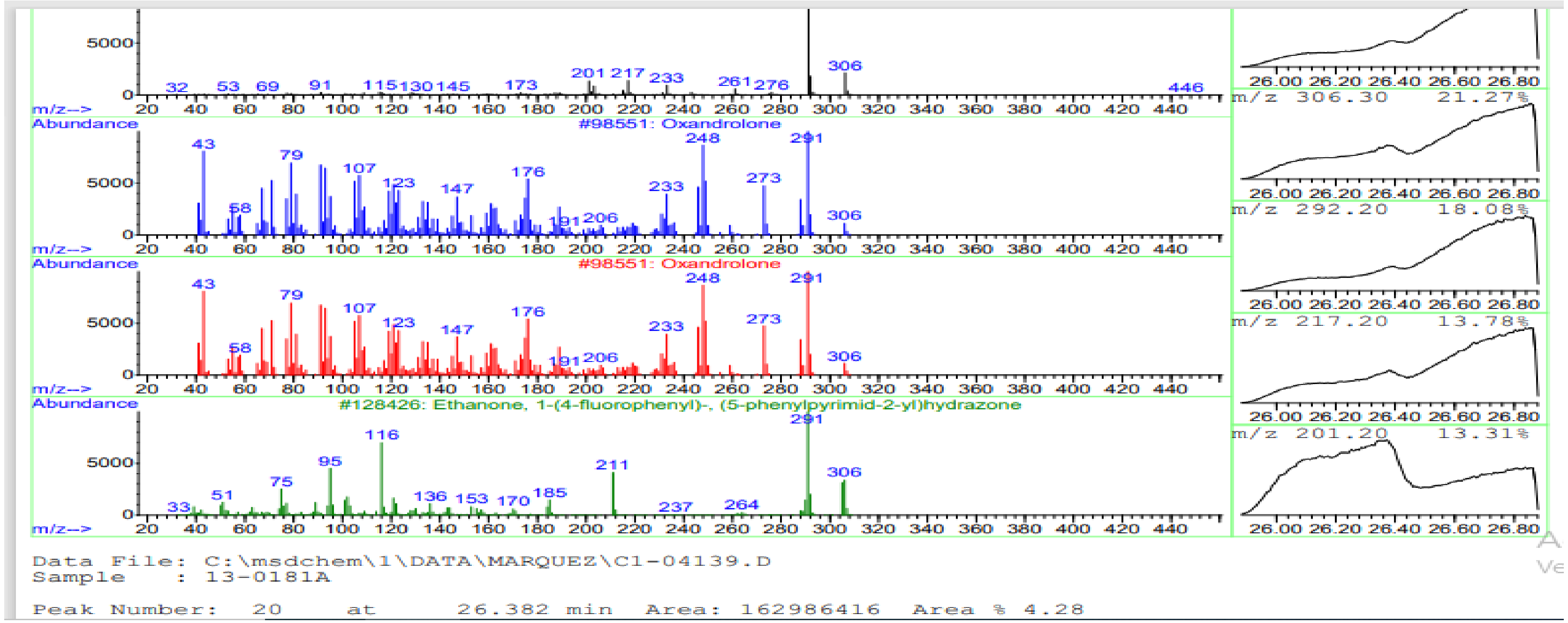

2-Chromancarboxylic acid, 6-amino-4-oxo-, ethyl ester

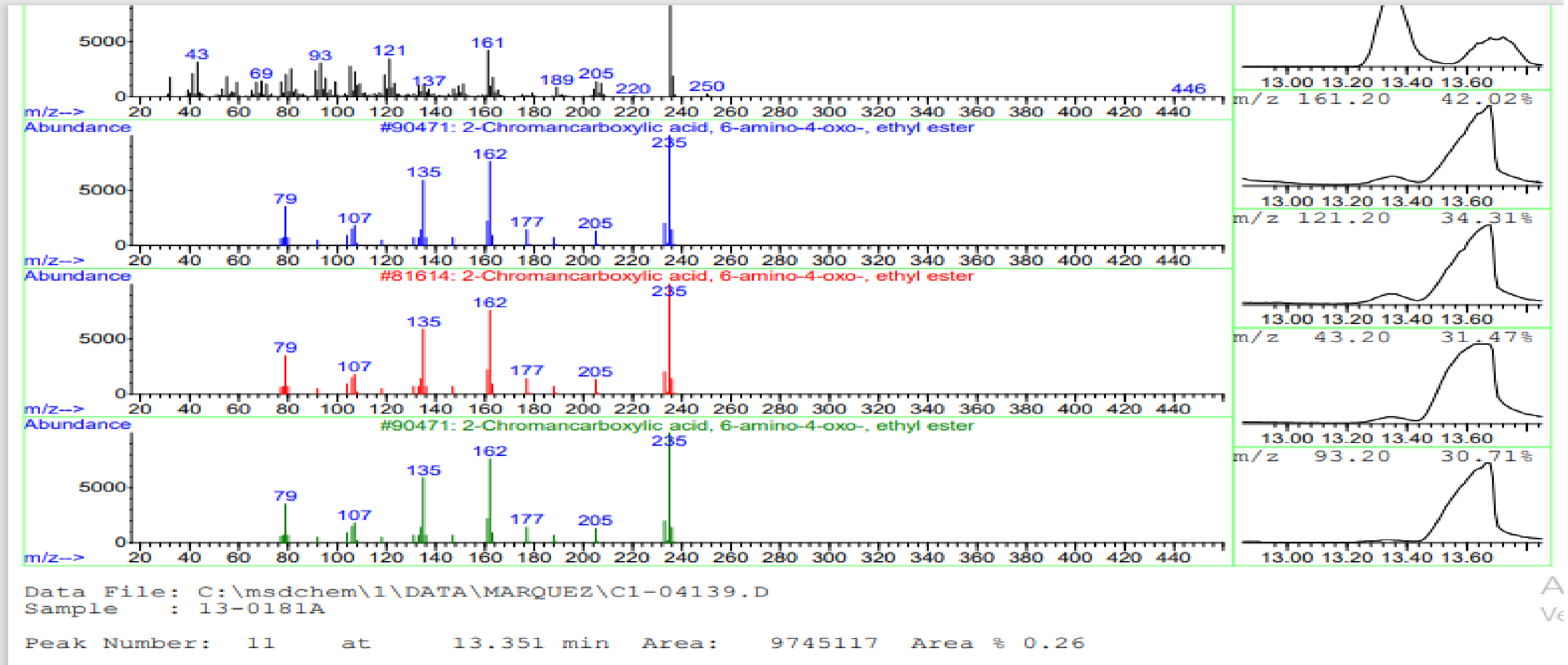

**Ethyl 5-oxo-4-(p-toluidino)-2,5-dihydro-3-furancarboxylate**	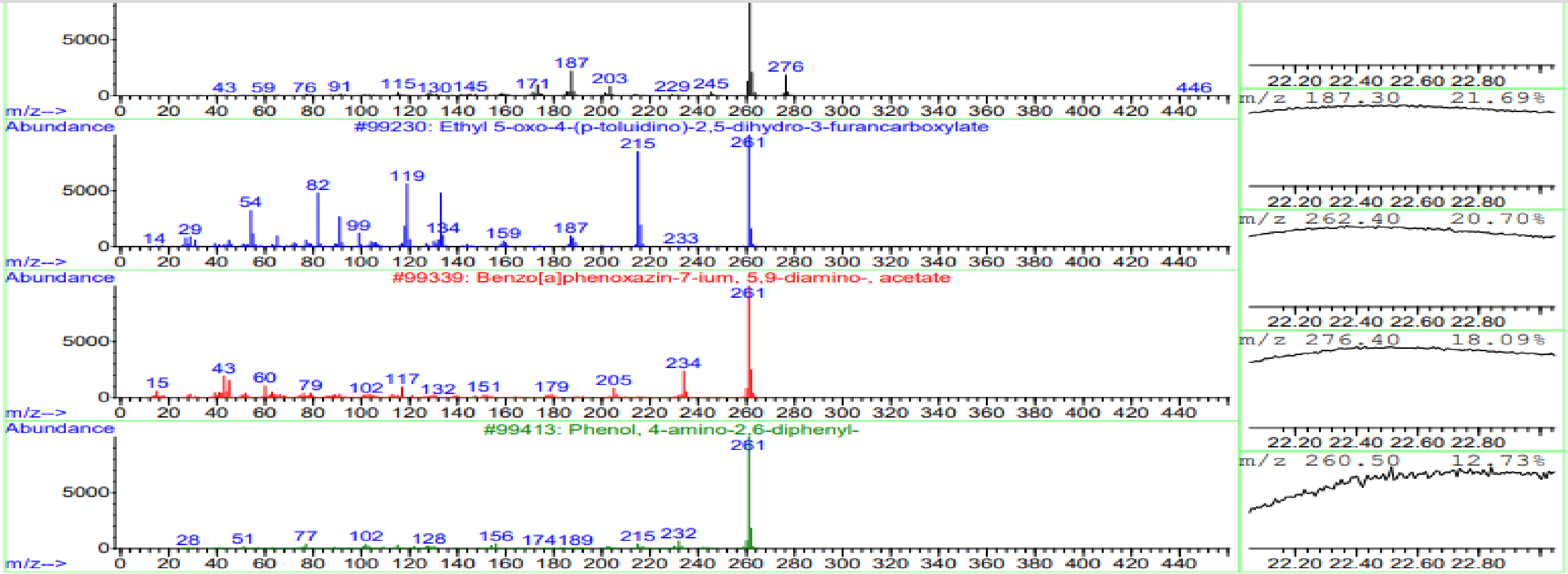

Data File: C:\msdchem\1\DATA\MARQUEZ\C1-04139.D
Sample : 13-0181A

Peak Number: 16 at 22.548 min Area: 2807481355 Area % 73.70

Pyrido[2,3-b]pyrimido[4,5-d]thiophen-4(3H)-one, 3-amino-9-methoxymethyl-2,7-dimethyl

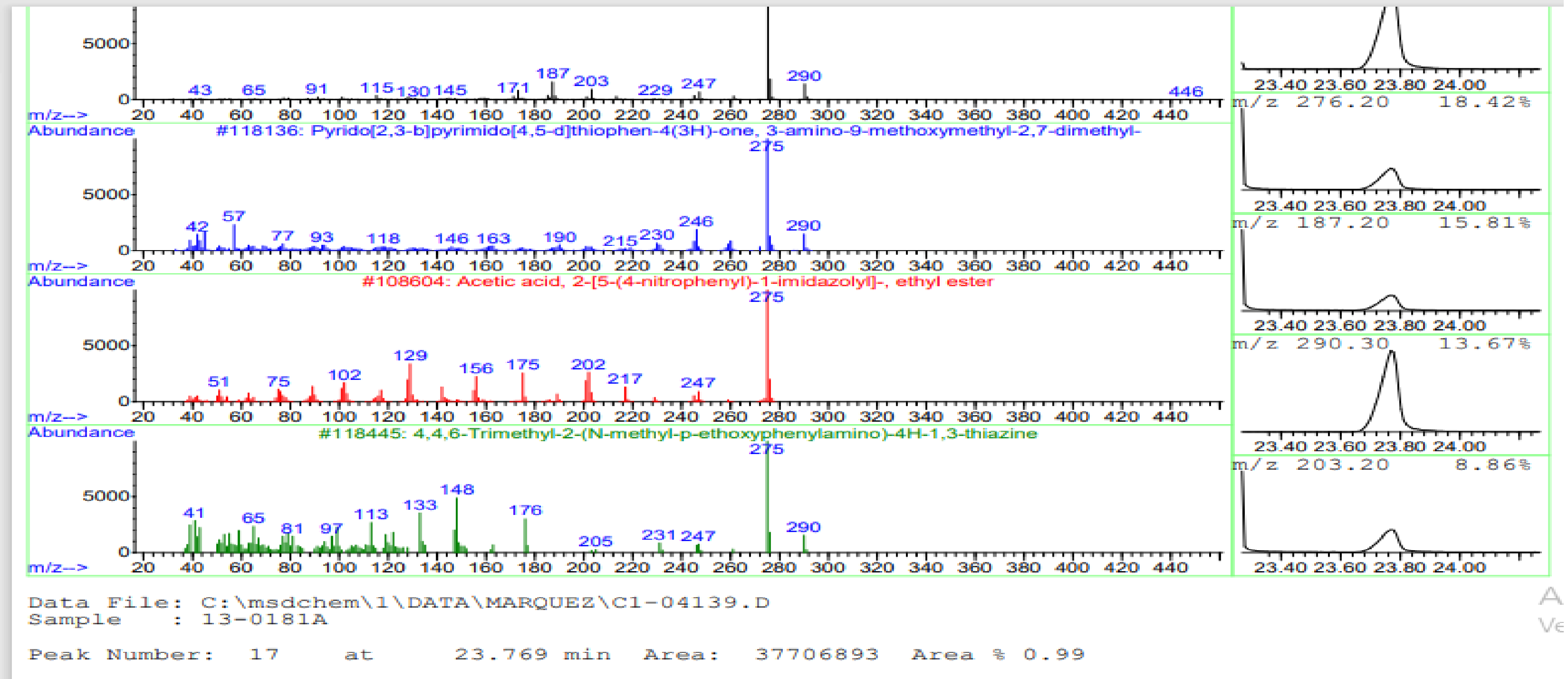

5,6,7-Trimethoxy-2,3-dihydrofuro(2,3-b) quinoline

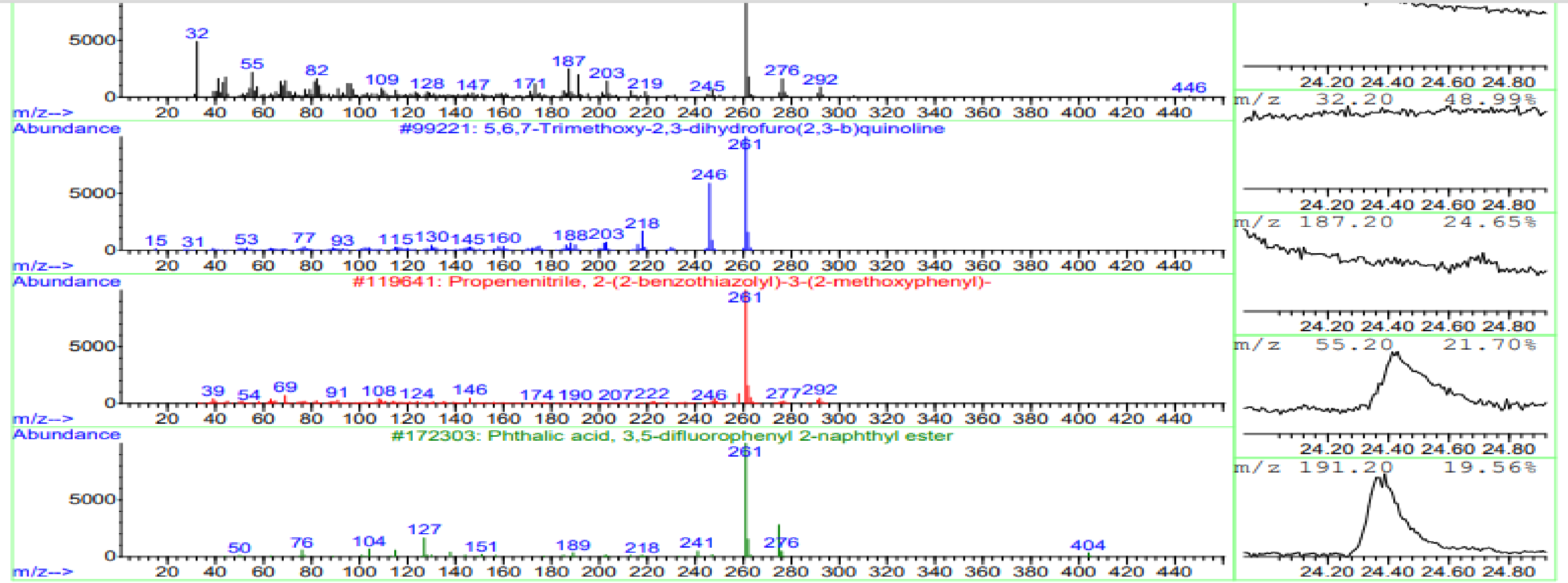

Data File: C:\msdchem\1\DATA\MARQUEZ\C1-04139.D
Sample : 13-0181A

Peak Number: 18 at 24.422 min Area: 1947881 Area % 0.05

Atherospermidine

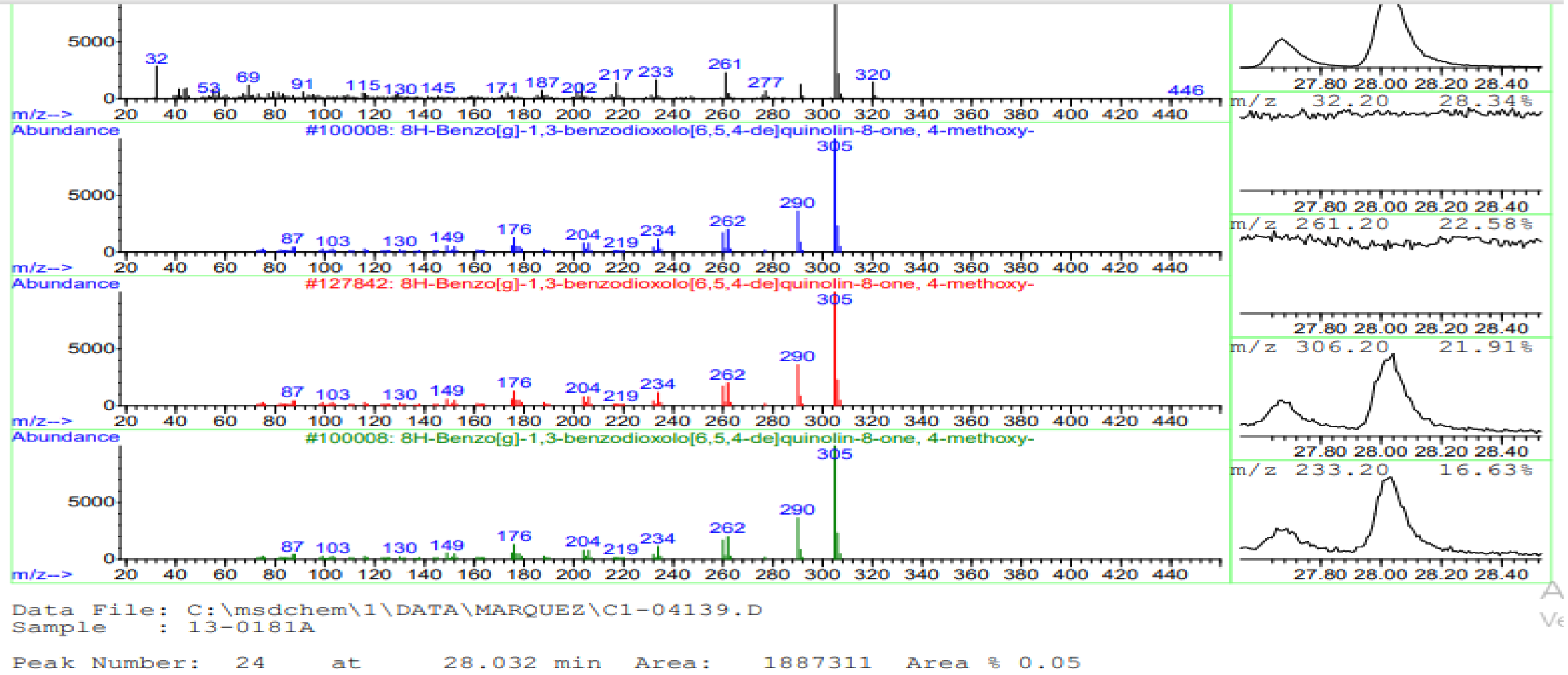

4,5,6,7-Tetrahydro-benzo[c]thiophene-1-carboxylic acid allylamide

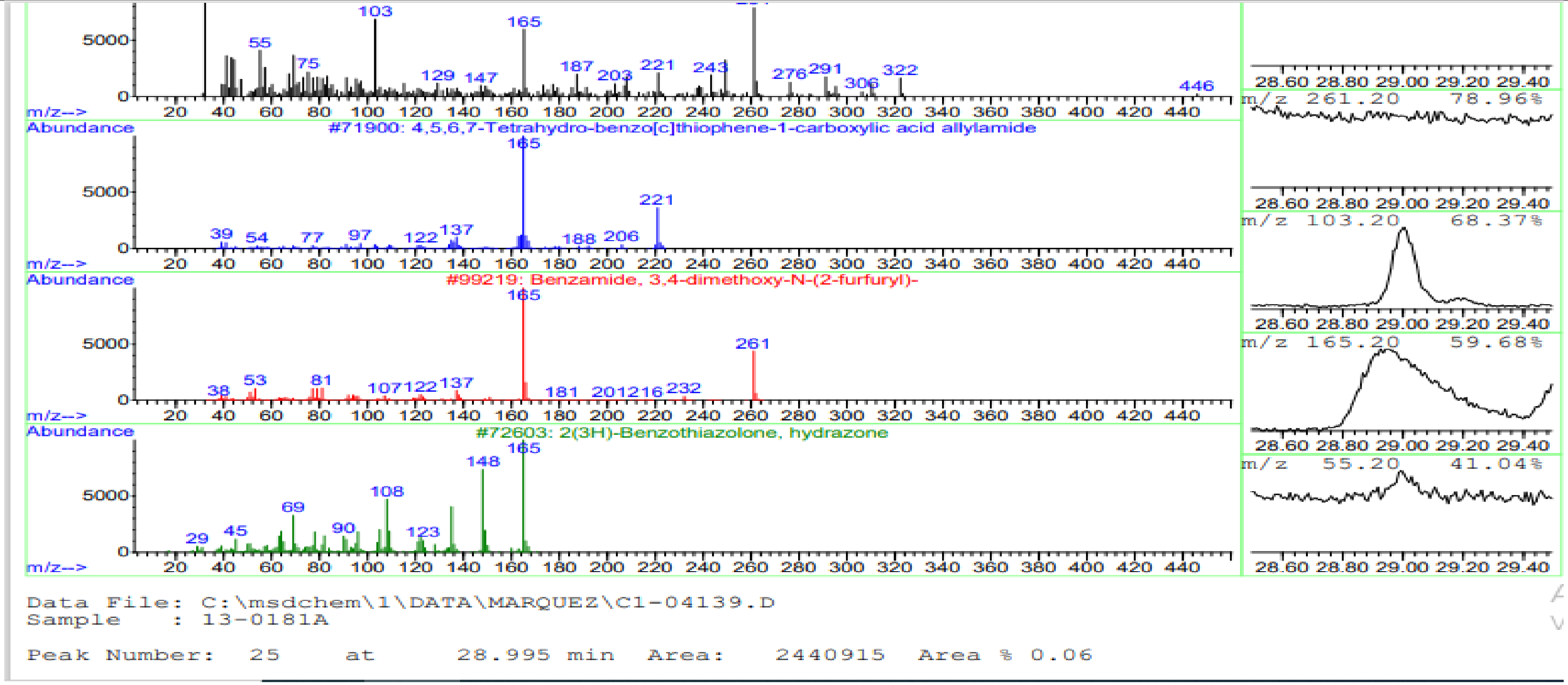

9-Phenylcarbazole

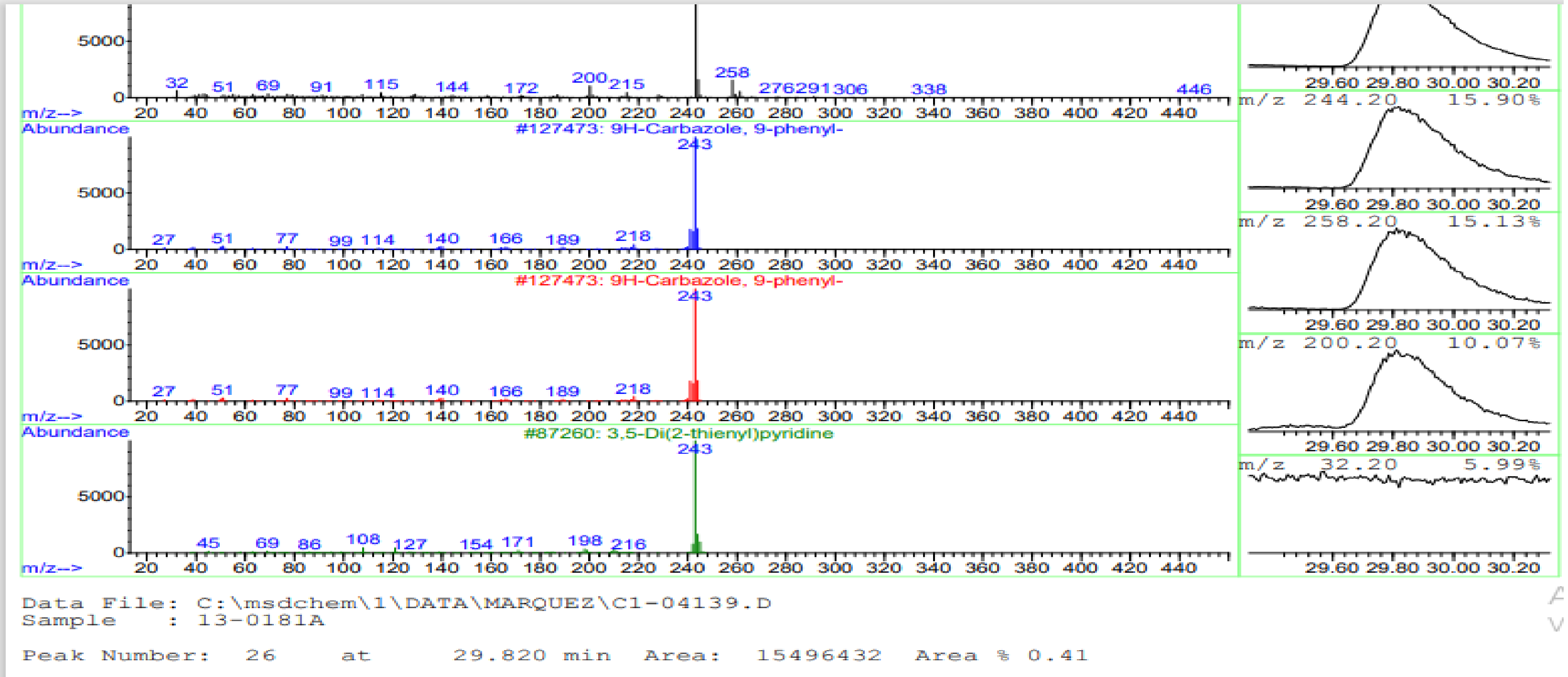

1-(1-Benzothien-2-yl)-N,N-diethylcyclohexanamine

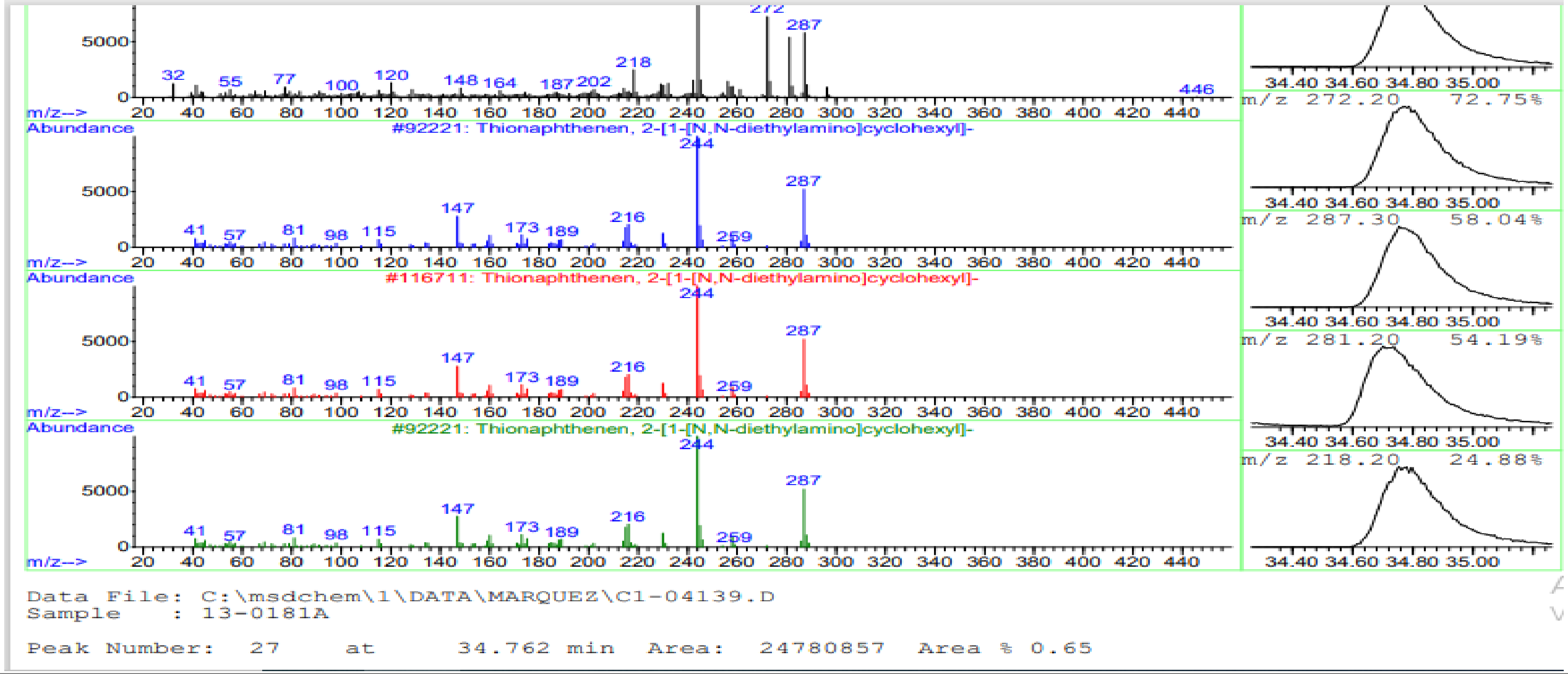

Methyl-methoxy-(1,1-difluoro-2,2-bis-trifluoromethyl-ethyl)amine

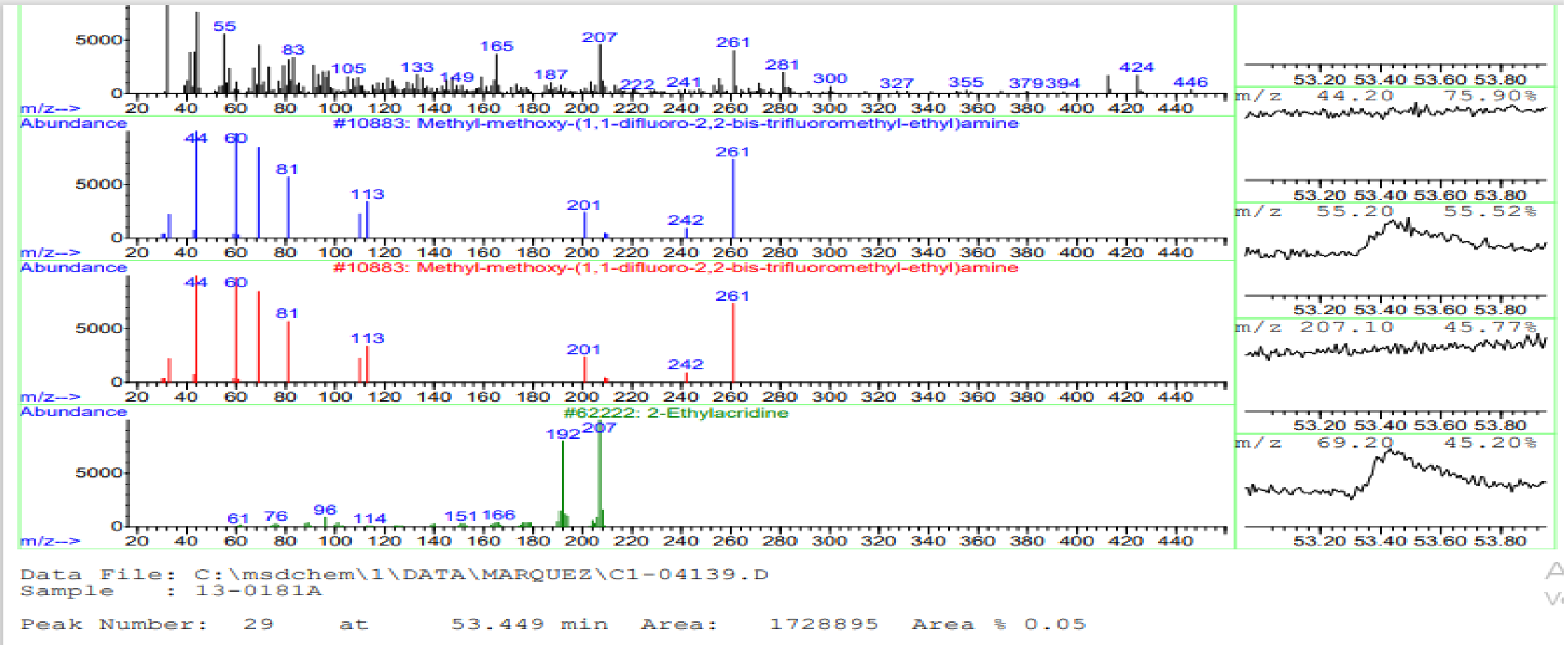

**Phenol, 4,4',4''-ethylidynetris**	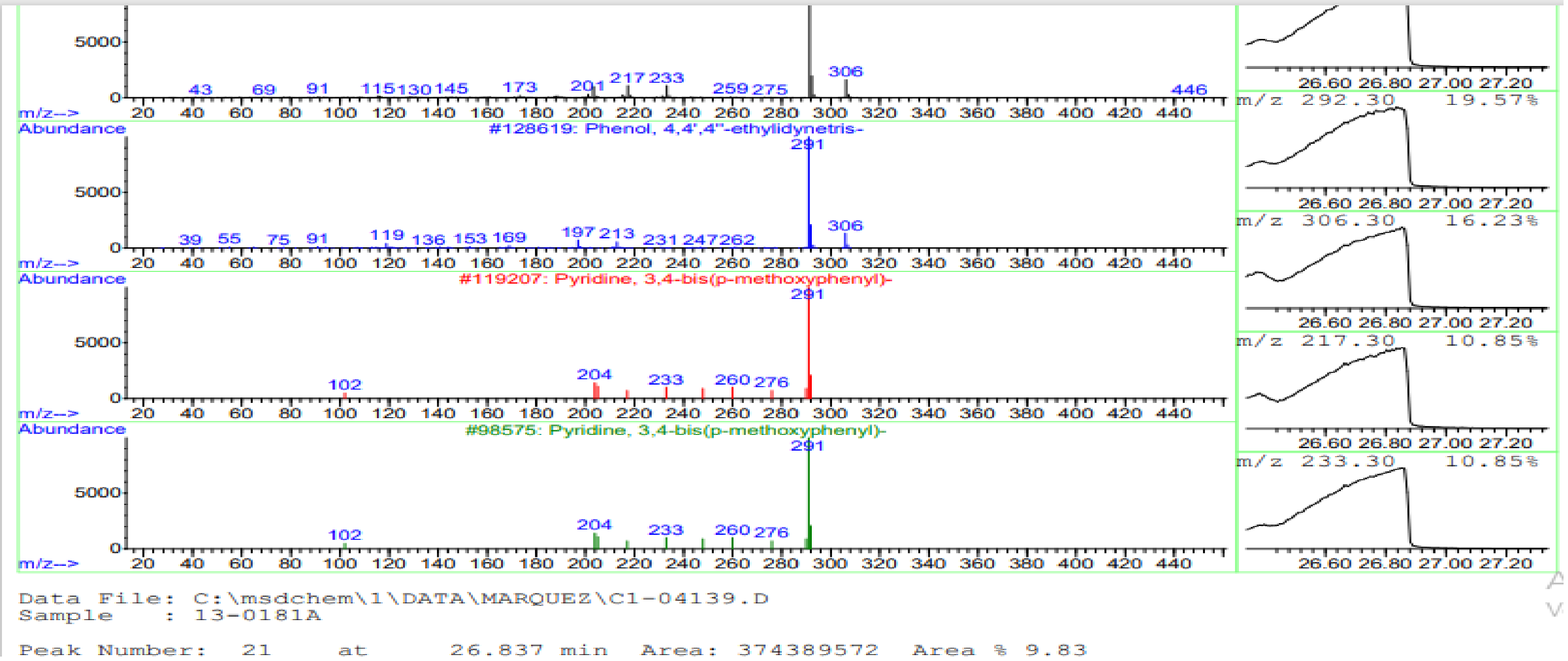

Ethyl Oleate

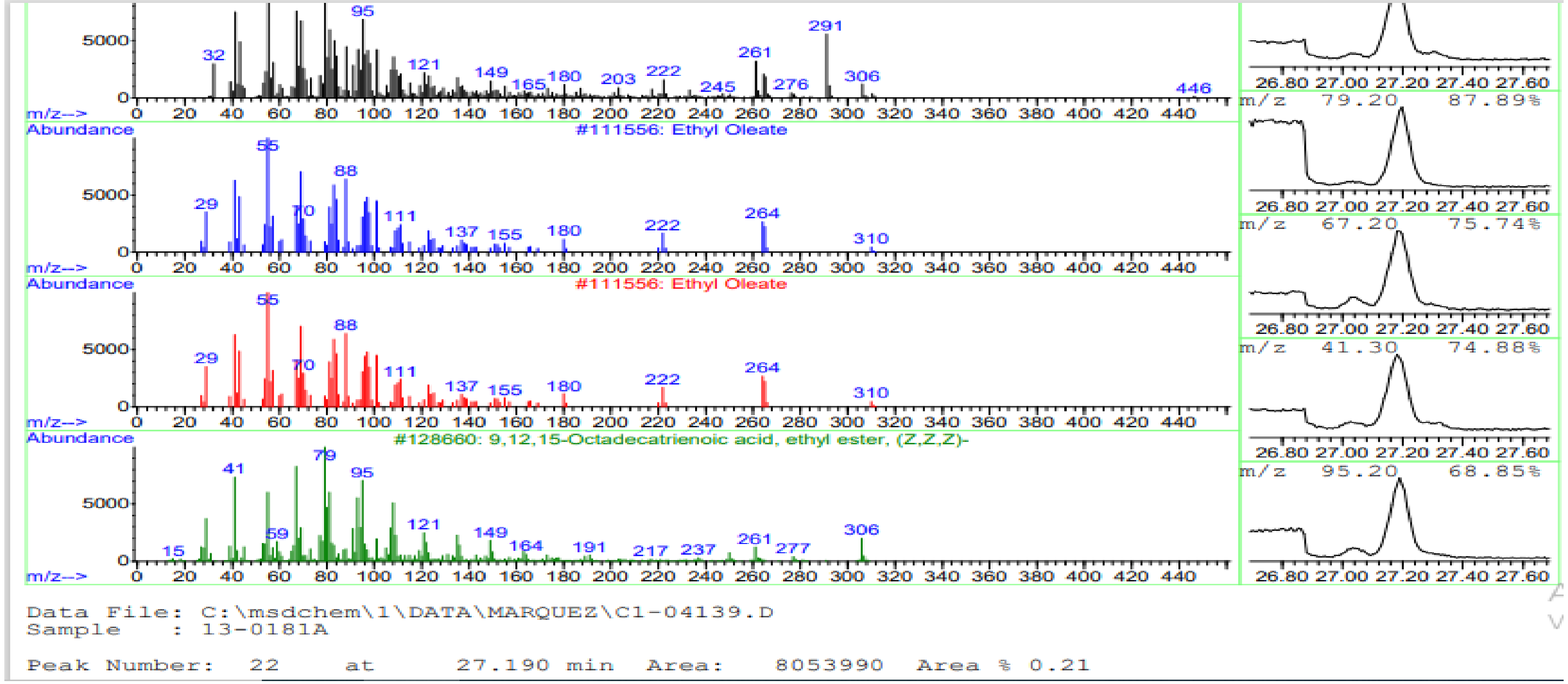

Figure S1. Fragmentation patterns of the mass spectra of the secondary metabolites present in the ethanolic extract of of *M. Americana* bark
