# Supplementary figures and images for: Ethnomedicinal Studies, Chemical Composition, and Antibacterial Activity of the Mammea americana L. Bark in the Municipality of Cértegui, Chocó, Colombia
Source: Adv Pharmacol Pharm Sci. 2022 Jan 19;2022:9950625. doi: 10.1155/2022/9950625 (PMC8791731; doi:10.1155/2022/9950625)

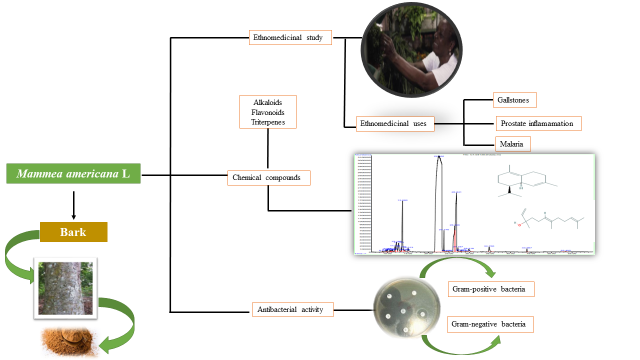

Supplement: Supplementary Materials — The following are available online. Figure S1. Fragmentation patterns of the mass spectra of the secondary metabolites present in the ethanolic extract of M. americana bark. [file 9950625.f1.zip › 9950625.f1/Graphical Abstract -1_ mammea americana _ Article (1).png]
